# Abstracts from the 17th annual conference of the Australia & New Zealand Academy for Eating Disorders (ANZAED 2019): Adelaide, Australia. 23–24 August, 2019

**DOI:** 10.1186/s40337-019-0272-0

**Published:** 2019-12-20

## Abstract

- co-facilitation with disciplines such as clinical nurse, psychologist and art therapist;
- the opportunity to complete weekly behavioural experiments at local cafes;
- longitudinal observation of behaviour change, impacting on quality of life

Challenges included:

- medical and psychological risk within group and during social eating outings
- interfering behaviours
- providing supportive meal therapy

This presentation will discuss these in more detail, including strategies used to address the challenges.

## Oral presentations

### O1 Combating disordered eating and poor body-image with the use of imagery Rescripting (IR) among body-dissatisfied young women

#### Yuan Zhou, Tracey Wade

##### Flinders University, Australia

###### **Correspondence:** Yuan Zhou (joanne.zhou@flinders.edu.au)

Imagery rescripting (IR) has been widely used to treat various mental health problem, however, little is known about its usefulness in eating disorders. A previous study suggested that a body-specific IR approach which rescripted negative body experience specific to disordered eating had a stronger effect on decreasing disordered eating and increasing body acceptance than a cognitive dissonance intervention among body-dissatisfied young women (Pennesi & Wade, 2018). The current study directly compared this approach with a general IR approach, which focused on general negative events that are not specific to the body or disordered eating. Young body-dissatisfied women who demonstrated an elevated risk of developing an eating disorder were randomly assigned to one of the four conditions, body-specific IR, general IR, psychoeducation and a control condition. All participants received a brief intervention in the lab, and those who were in the IR conditions practised IR for five minutes each day for a week. Disordered eating behaviours and body acceptance were measured at baseline and 1-week follow up. Preliminary results suggested that compared with body-specific IR, general IR resulted in larger effect size increases of body acceptance and decreases of disordered eating behaviours among participants. These findings provided preliminary support for general IR as a superior intervention to reduce risks for the development of an eating disorder.

### O2 Gut microbiota in Anorexia Nervosa and Atypical Anorexia – The effects of nutritional rehabilitation on gut microbiota: the ReGut study protocol

#### Madeline West^1^, Tetyana Rocks^1^, Fiona Collier^2^, Amy Loughman^1^, Felice Jacka^1^, Ruusunen Anu^1^

##### ^1^Food & Mood Centre, IMPACT SRC, Deakin University, Australia; ^2^Child Health Research Unit, Barwon Health

###### **Correspondence:** Madeline West (m.west@deakin.edu.au)

Emerging evidence has identified the importance of the gut microbiome (GM) in weight regulation, appetite, gut function, mood and anxiety, and behaviour, all of which are compromised in Anorexia Nervosa (AN) and Atypical Anorexia (AAN) patients. A key focus of inpatient rehabilitation programs for AN and AAN is weight restoration achieved through high-energy diets. However, the usual nutritional rehabilitation approaches may have adverse effects on the GM. Improved understanding of the potential role of the GM in AN/AAN would allow researchers to consider new and novel approaches, improving nutritional rehabilitation procedures. The ReGut (Recovering Gut) Study aims to understand the role of GM in these disorders by a) comparing the GM of AN and AAN patients and healthy controls and b) examining the GM composition before, during and after treatment in an inpatient unit. The associations between GM composition and patients’ symptomology will also be investigated. Patients (*n* = 40) with a diagnosis of AN or AAN will be recruited from a local inpatient eating disorder program. GM (stool samples), eating disorder behaviours, psychological and gastrointestinal symptoms and dietary intakes will be assessed at four time-points (admission, mid-treatment, discharge, and follow-up). Results from the ReGut study will provide insights into the role of GM in restrictive eating disorders and the impact of nutritional rehabilitation on GM.

### O3 How do adolescents in treatment for their eating disorder differ from those not seeking treatment?

#### Nora Trompeter^1^, Kay Bussey^1^, Phillipa Hay^2^, Chris Basten^1,3^, Jonathan Mond^4^, Mandy Goldstein^1,5^, Chris Thornton^1,6^, Gabriella Heruc^2,6,7,8^, Susan Byrne^9^, Stephen Touyz^10^, Alexandra Lonergan^1^, Scott Griffiths^11^, Deborah Mitchison^1,2^

##### ^1^Macquarie University, Centre for Emotional Health; ^2^Translational Health Research Institute, School of Medicine, Western Sydney University; ^3^Basten & Associates, Clinical Psychologists; ^4^Centre for Rural Health, University of Tasmania; ^5^Mandy Goldstein Psychology; ^6^Redleaf Practice; ^7^ANZAED; ^8^Appetite for Change; ^9^School of Psychological Sciences University of Western Australia, Perth, Australia; ^10^School of Psychology, University of Sydney; ^11^Melbourne School of Psychological Sciences, University of Melbourne

###### **Correspondence:** Nora Trompeter (nora.trompeter@mq.edu.au)

Introduction: Eating disorders frequently develop during adolescence, however few adolescents seek treatment. Demographics such as age and gender are most commonly associated with adolescents seeking treatment for eating disorders. However, there have been mixed findings in regards other demographic factors. For psychological factors, severity and distress have been linked with treatment-seeking (Forrest et al., 2016). The current study aimed to compare non treatment-seeking adolescents with an eating disorder to those who presented at eating disorder services for treatment.

Method: Data were used from a community sample of non-treatment seeking adolescents with an eating disorder (*n* = 1119) and a clinical sample of treatment-seeking adolescents (*n* = 115) in Australia. All participants completed self-report measures on demographics, eating disorder pathology (EDE-Q) and psychological distress (K10).

Results: Univariate analyses revealed that treatment-seeking adolescents had a lower BMI, higher SES, were older, and more likely to be female compared to adolescents not seeking treatment. However, when all demographic factors were considered, the two groups no longer differed on gender. Multivariate analysis revealed that weight/shape concerns were significantly higher whereas psychological distress was significantly lower among treatment-seeking adolescents compared to those not seeking treatment.

Conclusions: Demographic and psychological factors differentiated adolescents seeking treatment for their eating disorder from those not seeking treatment, thereby providing critical information for eating disorder services and early intervention programs.

### O4 Exploring the impact of age of menarche onset on eating disorders: The moderating role of personality traits

#### Sarah Giles^1^, Isabel Krug^1^, Roser Granero^2^, Susana Jimenez-Murcia^3^, Isabel Sánchez^3^, Nadine Riesco^3^, Fernando Fernandez-Aranda^3^

##### ^1^The University of Melbourne, Australia; ^2^Department of Psychobiology and Methodology, Autonomous University of Barcelona, Spain; ^3^CIBER Fisiopatologia Obesidad y Nutrición (CIBERobn), Instituto de Salud Carlos III, Barcelona, Spain

###### **Correspondence:** Sarah Giles (giless1@student.unimelb.edu.au)

Objective: To assess the relationship between the age of eating disorder (ED) onset and age of menarche across different ED subtypes, and to investigate whether personality traits moderated the relationship between menarche onset and ED severity.

Method: The sample consisted of 2568 treatment seeking ED patients [Anorexia Nervosa (AN), Bulimia Nervosa (BN), Binge-Eating Disorder (BED), Atypical-AN, Purging Disorder (PD), and Undefined Eating Disorder (UFED)] in Spain. Age of ED onset and age of menarche were assessed at intake by a trained clinician. ED symptom severity was measured by the total score of the Eating Disorder Inventory (EDI-2), and personality through the Temperament and Character Inventory-Revised (TCI-R).

Results: Across ED subtypes, the BED group reported higher mean differences in the number of years between ED onset and age of menarche, compared to the other ED subtypes, and the PD group reported higher mean differences than BN, AN, and Atypical-AN. Significant interactions for menarche-by-novelty seeking and menarche-by-reward dependence on ED severity were found, with novelty seeking was associated with lower ED severity, whereas higher reward dependence associated with higher ED severity. These findings were only observed in women with an earlier age of menarche.

Conclusion: The relationship between ED onset and age of menarche varies across ED subtypes. Furthermore, personality traits appear to interact with the age of menarche timing and ED severity.

### O5 Adolescents’ body idea internationalization at the intersection of sexual orientation and gender identity

#### Sarah M. Lipson^1^, Shirley B. Wang^1^, Jaquelyn L. Jahn^2^, Matthew K. Nock^1^

##### ^1^Department of Psychology, Harvard University, USA; ^2^Harvard T.H. Chan, School of Public Health, USA

###### **Correspondence:** Sarah Lipson (sarahlipson@college.harvard.edu)

A majority of the literature on sexual orientation and gender identity positions cisgender-heterosexual individuals as the participants. As such, this research may unintentionally reinforce heteronormative culture in a way that relegates gender and sexual minorities to the margins. Social body ideals, in particular, privilege cisgender-heteronormative bodies over those that do not fit this norm. Importantly, results obtained from this focus may not generalize to non-heterosexual non-cisgender populations, and no research to date has investigated internalized body ideals across strata of gender identity and sexual orientation. Therefore, in the current study we adopt an intersectional feminist framework to investigate adolescents’ internalized body image ideals across sexual orientation and gender identity. Participants (*N* = 454) were recruited online over social media forums as part of a larger study. Analyses that compare self-reported body ideal internalization by sexual identity and gender orientation using one-way ANOVAs are currently underway. These results will shed light on social body ideal internalization across gender identity and sexual orientation in adolescents. Findings from this study can increase our understanding of how body image ideals manifest for these populations, which may enhance eating disorders prevention and intervention efforts for individuals across different identities.

### O6 Collaborative community planning: The WA Eating Disorders Outreach & Consultation Service (WAEDOCS)

#### Janet Fountaine^1^, Fintan O’Looney^2^, Melissa Edwin^3^, Anthea Fursland^1^, Lisa Miller^1,4,5^, Kathy Logie^6^

##### ^1^WAEDOCS, Australia; ^2^Hollywood Private Hospital; ^3^WA Eating Disorders Outreach & Consultation Service; ^4^University of Western Australia; ^5^Sir Charles Gairdner Hospital, Perth; ^6^Hollywood Clinic

###### **Correspondence:** Janet Fountaine (janet.fountaine@health.wa.gov.au)

Background: There is no public specialist multi-disciplinary service for people with eating disorders in Western Australia (WA). The only public specialist outpatient service, the Centre for Clinical Interventions (CCI), is a psychology service, and is in Perth. In 2017, the WA Eating Disorders Outreach & Consultation Service (WAEDOCS) was created to support, mentor, and guide clinicians throughout WA. Aims: We wanted to facilitate improved communication between all clinicians involved in an individual’s care.

Method: WAEDOCS developed a Collaborative Community Plan (CCP), identifying best practice tasks and facilitating communication links between the virtual treatment team.

Results: Clinicians reported improved communication and shared knowledge of e.g., a person’s latest weight, their eating patterns, attendance at appointments, medical stability.

Conclusion: The CCP facilitates communication, reduces splitting among the team and provides a safety net for people with eating disorders.

### O7 Solving the supervision dilemma for dietitians working in eating disorders - Innovative Programmes from a state wide advisory service

#### Amanda Davis^1^, Amy Davis^2^

##### ^1^Queensland Eating Disorder Service, Australia; ^2^Child & Youth Mental Health Service, Eating Disorder Programme

###### **Correspondence:** Amanda Davis (amanda.davis@health.qld.gov.au)

Provision of dietetic interventions in eating disorder (ED) treatment is an advanced practice area and high level clinical supervision is recommended. Historically dietitians have not engaged in paid supervision, plus dietitians with adequate experience in EDs and supervisory training are often over-subscribed. Lack of supervision may contribute to dietitians feeling overwhelmed or unsupported in this workload and inadequate knowledge of evidence-based practice. In 2018 QuEDS launched two innovative programmes to improve access to supervision and support to dietitians within Qhealth. 1. Facilitated-Peer Group Supervision (QuEDS f-PGS) for Dietitians (via video conference) with an educational component - closed group of 10, monthly 90 min sessions for 1 year. Pre, 6 month and post completion surveys indicate increased levels of competence/confidence to work in this challenging field. In future, a generic QuEDS f-PGS package will be developed to facilitate roll out from other health professionals within QuEDS and other specialist ED services. 2. Dietitians Knowledge Pathway (QuEDS DKP) – desktop resource, targeted at dietitians and providing local knowledge. The user is guided through treatment guidelines, pathways, seminal references, online learning opportunities, professional associations, local resources etc. User determines pace and depth of learning. Pre- and post-completion quality surveys indicate users feel more confident and better informed. In future, the QuEDS DKP will be available as an online resource.

### O8 Outcomes of a regional day program treatment service for eating disorders

#### Emma Gallagher (HNELHD-EDDPayprogram@hnehealth.nsw.gov.au)

##### James Fletcher Hospital Eating Disorders Day Program, Australia

The Eating Disorders Day Program in Newcastle supports clients with Anorexia Nervosa, Bulimia Nervosa and Binge Eating Disorder. It is a semi-closed group format running for up to 12 weeks, for up to eight clients at a time. Clients receive meal support for the provided breakfasts, morning teas and lunches, as well as CBT-E and DBT group therapy. The program also has a strong focus on sensory processing and mental health rehabilitation. In addition to weekly weighing, clients regularly complete measures of eating disorder symptomatology (EDEQ), body image, emotion regulation, quality of life, self-compassion, valued living, and depression anxiety and stress. The presentation will outline client experiences of the program, changes to the program content and delivery since inception, as well as the effectiveness of the program for each clinical group at discharge. Key effectiveness markers include weight change, Global EDEQ score, and quality of life domains. Correlational outcomes will be presented for the last four years, highlighting which variables are linked to positive outcomes in weight restoration and changes in eating disorder symptomatology. Future directions for the service will be discussed, including navigating changes to the district service plan for eating disorder treatment, and ongoing partnerships with private practitioners.

### O9 Bringing the voice of carer lived experience to the clinical table

#### Belinda Caldwell^1^, Michelle Roberton^2^

##### ^1^Eating Disorders Victoria; ^2^CEED Victoria

###### **Correspondence:** Belinda Caldwell (belinda.caldwell@mh.org.au)

Bringing the voice of carer lived experience to the clinical table is an emerging role within eating disorders services. The Victorian Centre of Excellence in Eating Disorders has been at the forefront in modelling to services and clinicians the value of carer lived experience in informing clinical review of cases, training and education development, service and resource development, along with the value of lived experience as a change agent in treatment.

This presentation will provide an overview of the role, how it is operationalised and evaluation results. In particular, we will focus on the carer consultation service that CEED offer to CAMHS/CYMHS services. The carer consultation service brings together the carer consultant, a CEED senior clinician, the local CAMHS team and the family in treatment, in a single session approach focused on addressing stumbling blocks to progress in treatment. We will present data on its effectiveness as both a change agent and a clinical learning tool, as well as discuss the emerging themes from the carer consultations we have undertaken.

As we recognise the key role families and carers play in achieving effective treatment outcomes, carer consultants or peer support workers will become a critical workforce within the eating disorders sector. Models for how best to utilise this workforce are needed to inform this evolution.

### O10 A whole of health system service reform program to embed the identification, access and treatment of eating disorders within mainstream health services: five years on what has been achieved?

#### Danielle Maloney^1,2,4^, Sarah Maguire^1,2^, Michelle Cunich^3,1^, Ang Li^3,1^, Jane Miskovic-Whealtey^1^, Ian Caterson^3,1^, Teresa Anderson^4^, Sloane Madden^5,1,6^, Andrew Wallis^5^, Joanne Titterton^5^, Janice Russell^1^, Bridget Mulvey^7^, Lyn Chiem^8^

##### ^1^Sydney University; ^2^InsideOut Institute; ^3^Boden Institute; ^4^Sydney Local Health District; ^5^The Sydney Children’s Hospital Network; ^6^The Redleaf Practice; ^7^RPAH/tertiary; ^8^Royal Prince Alfred Hospital

###### **Correspondence:** Danielle Maloney (danielle.maloney@sydney.edu.au)

NSW Health embarked 5 years ago on an ambitious program of health system reform to address the lack of access to treatment for people with eating disorders in NSW. This large-scale service and workforce development program, affirming eating disorders as part of the core business of the health system, began a process of transforming eating disorder treatment in every local health district across NSW. The program received initial funding of $17.6 million over 5 years and the InsideOut Institute for Eating Disorders, based at the University of Sydney, was funded to lead the reform in partnership with the two specialist hubs for child & adolescent and adult treatment of eating disorders. The program involves broad scale engagement at all levels from Chief Executive to clinician on the ground; policy reform in every setting covering community, emergency department and hospital treatment; building of treatment pathways and models for evidence-based programs; and large-scale workforce development to de-stigmatise and change health worker practice. Pre and post NSW Health data over the 5 years indicates significant increases in access and treatment of eating disorders across all service settings. In some areas up to a 50% increase was observed. Pre and post data examining clinician skills and willingness to treat people with eating disorders also showed significant increases across all NSW.

### O11 Strengthening the system of care for adults experiencing eating disorders: A public mental health service initiative

#### Rachel Knight^1^, Beth Shelton^2^, Alexandra Hillman^3^, Sarah Trobe^1^

##### ^1^Victorian Centre of Excellence in Eating Disorders; ^2^NEDC; ^3^NorthWestern Mental Health

###### **Correspondence:** Rachel Knight (rachel.knight@mh.org.au)

In Victoria, eating disorder (ED) treatment for adults in the public health system is often only available through specialist services. However, adults may be unable to access these services due to geographical constraints, comorbidity or level of risk.

NorthWestern Mental Health (NWMH) is an adult public mental health (MH) service in Melbourne. Commencing in 2017, NWMH collaborated with the Victorian Centre of Excellence in Eating Disorders to enhance the generalist community MH response for EDs. The objectives of the initiative included establishing seven eating disorder clinician (EDC) roles, a workforce development strategy and intake process changes.

Utilising an analysis framework adopted from Gervais (2008), qualitative data was obtained from EDCs. Data was collected at the beginning of the initiative and at 10 month follow up. Initially, EDCs identified key opportunities in increasing the availability of community-based support and improving intake pathways. Key needs included clarifying the EDC role functions and workforce training.

Data collected at the second time point showed that clinicians were more confident to support people experiencing an ED and clearer referral pathways were established. EDCs also identified a need to strengthen links with community services such as GPs and for improved service policies. This research suggests a multifaceted service change initiative can result in an enhanced provision of support for eating disorders in generalist public MH services.

### O12 The effectiveness of an ACT-based intervention in the management of disordered eating

#### Katie Babbott (katie.b@hotmail.co.nz)

##### University of Waikato, New Zealand

‘Disordered eating’ refers to patterns of thoughts and behaviour that are maladaptive, and centred around food, weight, and eating. For individuals who engage, the risk of progression to a clinically significant eating disorder is high. This research evaluated the efficacy of a low-cost intervention which is publicly available and may be used to address high rates of diagnosis and demand for services. The efficacy of Acceptance and Commitment Therapy (ACT) in treating a broad range of disorders has been well-established in empirical literature. The present study utilised a non-concurrent multiple baseline design to evaluate the efficacy of a self-help ACT workbook in managing non-clinical disordered eating. Participants were asked to complete pre and post-intervention measures of acceptance, valued living, disordered eating pathology, and psychopathology. The book, ‘Get Out of Your Mind and Into Your Life’ was used for this study. Following a two-week baseline, seventeen participants worked through select chapters in the book over the course of six weeks. Upon completion, acceptance and quality of life ratings showed improvements at a trend level, and disordered eating pathology significantly decreased. All improvements were maintained at follow up. The results of this research support the hypothesis that self-help interventions can be a useful for mitigating subclinical disordered eating, and suggest that further research is warranted into the development of ACT based interventions.

### O13 Introducing DBT as a treatment for binge eating and bulimia

#### Tanya Gilmartin (tanyalgilmartin@gmail.com)

##### RO-DBT Australia

Dialectical Behaviour Therapy (DBT) was initially designed for the treatment of Borderline Personality Disorder. Since the publication of the original treatment manual in 1993, DBT has been utilised as an effective treatment protocol for disorders characterised by cognitive, emotional, behavioural and interpersonal dysregulation. One adaptation that has been published is DBT as a treatment for Binge Eating and Bulimia (DBT-E). The DBT-E treatment protocol is based on an emotion dysregulation model of problem eating, and provides clinicians with additional commitment strategies, mindfulness strategies and relapse prevention strategies to assist clients to obtain and maintain abstinence from binge eating and purging behaviour. This presentation will provide delegates with an overview of the theoretical base for DBT-E, as well as an overview of commitment and mindfulness skills that are designed to target problem eating behaviour.

### O14 Community based meal support: An innovative program in the treatment of eating disorders

#### Belinda Chelius, David Langford

##### Eating Disorders Queensland, Australia

###### **Correspondence:** Belinda Chelius (admin@edq.org.au)

The Community Table is a community-based meal support program run by Eating Disorders Queensland and co-facilitated with the Queensland Eating Disorder Service. The Community Table was established to help fill the gap in recovery, when an individual transitions to community-based treatment, but requires continued support in meal adequacy and connected eating. It was hypothesised that providing a face-to-face meal support program, with online resources, would help facilitate ongoing recovery from an eating disorder in the community.

The Community Table runs weekly for 5 weeks and aims to support the individual to work towards personal goals around nutrition and connected eating, rediscover their relationship with food, explore skills to manage intrusive thoughts and feelings that accompany eating and to try new activities that may be helpful at pre and post meal times.

Pre and Post data is collected from participants to evaluate the effectiveness of the program. As the program is in its preliminary stages, quantitative data is continuing to be collected and evaluated. Qualitative feedback has also been collected from a sample of 17 participants. The emerging themes of the program include the importance of connection in recovery. The Community Table program led to an increased sense of connection and it was beneficial to receive support from Eating Disorders Professionals when working towards personal goals around nutrition and connected eating.

### O15 The impact of outpatient nutrition and dietetic intervention on clinical outcomes for adults with an eating disorder: A systematic review and assessment of evidence quality

#### Caitlin M McMaster^1^, Susan Hart^1,2^

##### ^1^Boden Collaboration for Obesity, Nutrition, Exercise and Eating Disorders, University of Sydney, Australia; ^2^St Vincent’s Hospital, Sydney

###### **Correspondence:** Caitlin M McMaster (caitlin.mcmaster@sydney.edu.au)

Although reviews of medical, pharmacological and psychological treatments for patients with an ED have been conducted, no reviews have examined literature regarding the impact of outpatient nutrition and dietetic intervention on patient outcomes. Systematic searches of Medline, PsychINFO, CINAHL, AMED and Embase were conducted for studies examining the impact of individual or group outpatient nutrition and dietetic treatment, education or counselling on weight, ED behaviours and nutritional outcomes in adults with anorexia nervosa (AN), bulimia nervosa (BN) or binge eating disorder (BED). The Grading of Recommendations Assessment, Development and Evaluation (GRADE) framework was used to assess the quality of evidence for the impact of nutrition and dietetic intervention on reported clinical outcomes for each ED diagnosis. Twelve papers reporting on 11 randomized controlled trials met the inclusion criteria. Application of the GRADE framework showed a low quality of evidence for the impact of nutrition and dietetic intervention on all rated outcomes for AN (treatment completion, weight change, remission rate), BN (treatment completion, frequency of binge eating and compensatory behaviours, nutritional outcomes) and BED (weight change, treatment completion, hunger and satiety). The results of this systematic review and strength of evidence assessment indicate the need for further research to adequately assess the impact of nutrition and dietetic intervention on a range of clinical outcomes for patients with AN, BN and BED.

### O16 The nature of the therapeutic Alliance between nurses and consumers with anorexia nervosa in the inpatient setting

#### Joel Zugai, Jane Stein-Parbury, Michael Roche

##### University of Technology Sydney, Australia

###### **Correspondence:** Joel Zugai (joel.zugai@uts.edu.au)

Background: Although therapeutic alliance is associated with good outcomes, relationships between nurses and consumers with anorexia nervosa are often strained. The nature of the therapeutic alliance in the inpatient setting is relatively under researched.

Aim: To understand the nature of the therapeutic alliance between nurses and consumers with anorexia nervosa in the inpatient setting.

Methods: An explanatory sequential mixed methods approach was employed. Surveying both consumers and nurses, the quantitative phase measured the strength of the therapeutic alliance, and other aspects of the ward context. The results of these measures were qualitatively explored through semi-structured interviews with both nurses and consumers.

Results: There was a contrast between views of the therapeutic alliance between consumers and nurses, with consumers rating the alliance relatively poorly. Consumers valued nurses who engaged with interpersonal tact, and nurses who treated them as an individual rather than an illness. Whilst nurses could not be overly authoritative or coercive, nurses maintained professional boundaries in order to preserve the therapeutic power differential.

Conclusion: The therapeutic alliance was dependent on the perceived therapeutic merit of the power differential between nurses and consumers. In a therapeutic alliance, consumers trusted that nurses genuinely cared for them, and felt safe in investing in a new concept of wellbeing. A maternalistic style of nursing emerged as an expedient approach.

### O17 Barriers and facilitators to treatment-seeking for eating disorders: Exploration of the reach out and recover online support program

#### Siân McLean^1^, Belinda Caldwell^2^, Michelle Roberton^3^

##### ^1^Victoria University; ^2^Eating Disorders Victoria; ^3^Victorian Centre of Excellence in Eating Disorders & Private Practice

###### **Correspondence:** Siân McLean (sian.mclean@vu.edu.au)

Objective: The treatment gap in eating disorders is well known. This study aimed to report on the treatment seeking of consumers accessing Reach Out And Recover (ROAR), an online resource to enhance motivation and confidence to seek treatment, and to explore correlates of barriers and facilitators to treatment-seeking.

Method: Participants were 200 ROAR users aged 15 to 60 plus (93.5% female). Data consisted of responses to questions embedded in ROAR regarding attitudes to treatment, intentions to seek treatment, eating disorder symptoms, and impact on health. Results: Most participants (86.0%) were not receiving treatment, despite high levels of eating disorder symptoms. However, the majority (82.6%) indicated they planned to seek treatment. A personalised general practitioner report to facilitate treatment-seeking was downloaded by more than half of participants (52.9%). Intentions to seek treatment was positively associated with motivation and confidence to achieve change, greater binge eating frequency, and greater impact of eating disorder symptoms on well-being.

Conclusion: The ROAR website was utilised by consumers with high levels of eating disorder symptoms who were not otherwise receiving treatment. Nonetheless, participants had strong intentions to seek treatment, and encouragingly, many downloaded resources to facilitate action towards treatment. Findings suggest that focusing on motivation and confidence to change and symptom impact on wellbeing, rather than stigma or eating disorder symptoms, may enhance treatment-seeking.

### O18 Reducing stigma using first-person accounts of eating disorders: A transportation theory approach

#### Bethany Morris^1^, Joanna Doley^1,2^, Arthur Stukas^1^

##### ^1^La Trobe University, Australia; ^2^Victoria University

###### **Correspondence:** Bethany Morris (bthnmorris@gmail.com)

Background: Transportation theory suggests that texts which transport readers by encouraging emotional engagement (e.g. through descriptiveness, and protagonist identification) can induce positive attitude change towards stigmatised groups (Green & Brock, 2000). Transportive ED memoirs may reduce ED stigma; however, some research suggests that ED memoirs could unintentionally glamourise EDs or increase symptoms. We examined whether memoirs could reduce stigma using a transportation theory framework, and monitored for unintended harm.

Method: Online participants (*N* = 195 women) were randomly assigned to one of four brief ED memoirs varying descriptiveness and protagonist similarity (gender). Participants then reported levels of transportation, emotional responses, stigma (social distance, responsibility attributions), glamourisation, and ED symptoms.

Results: Narrative descriptiveness demonstrated indirect effects through increased transportation on improved social distance (b = .04, se = .02, 95%CI [0.00, 0.10]) and reduced personal responsibility (b = −.02, se = .01, 95%CI [− 0.05, − 0.00]) but also on higher ED symptoms (b = .09, se = .04, 95%CI [0.01, 0.19]). Transportation did not vary by protagonist gender.

Discussion: Descriptive ED memoirs that transport readers may show promise for reducing ED stigma. However, future research needs to examine whether vulnerable readers are more likely to feel transported by descriptive memoirs.

### O19 “Well you’re obviously not anorexic!” The barriers people struggling with eating disorders face when seeking help and how treatment experiences may be improved

#### Roma Watterson^1^, Janet Carter^1^, Jennifer Jordan^2^, Lois Tonkin^1^

##### ^1^University of Canterbury, New Zealand; ^2^University of Otago, Christchurch

###### **Correspondence:** Roma Watterson (roma.watterson@pg.canterbury.ac.nz)

Despite potentially severe impacts, only a proportion of people experiencing an eating disorder (ED) receive treatment and, for those who do, there is still considerable room for improvement in treatment efficacy. This study aimed to establish a better understanding of factors that may be important in treatment and recovery, from the perspective of New Zealand women with a lifetime history of an ED. Qualitative research comparing Anorexia Nervosa (AN), Bulimia Nervosa (BN) and Binge Eating Disorder (BED), from the perspective of people with an ED, is extremely limited. In-depth, semi-structured interviews were carried out with 18 women with a lifetime history of an ED. The sample included AN, BN and BED, and recovered and non-recovered participants. Thematic analysis of transcribed interviews followed guidelines proposed by Braun and Clarke. Any differences in the presence of themes between diagnostic subtypes were then investigated. Participants discussed barriers they encountered to treatment and recovery, including obstacles they had to overcome to reach out for help, and responses they received when they did. Positive and negative treatment experiences, and ways in which participants believed treatment could be improved were identified, with some diagnostic variations evident. Incorporating feedback from consumers has the potential to improve the accessibility and uptake of and engagement with treatment, which may thereby contribute to better outcomes for people with EDs.

### O20 Peer Mentor program benefits in eating disorder recovery

#### Emma Trappett, Emily Stanley, Belinda Chelius

##### Eating Disorders Queensland, Australia

###### **Correspondence:** Emma Trappett (admin@edq.org.au)

The Peer Mentor Program at Eating Disorders Queensland is a collaborative program that aims to address identified gaps in services for people recovering from eating disorders, by providing a comprehensive recruitment and training program for peer mentors and volunteers that includes, building successful mentoring relationships, risk management and self-care. A comprehensive evaluation strategy is run concurrently with the program.

From years 2015–2018, pre and post data from the Depression Anxiety Stress Scale (DASS21) were analysed and collected over a six-month period whilst involved in PMP. A sample of 21 mentee’s were included in the analysis. Overall, 76% of participants experienced an improvement or no change across all three sub-measures- depression, anxiety, stress. Research findings showed a statistically significant decrease in anxiety scores from pre to post measure (9.52% of participants worsened, 38.10% had no change and 52.38% improved). Further t-test analysis showed an improvement in scores for depression and stress however is was not statistically significant. Concurrently, qualitative data has continued to be collected from mentors and mentee’s throughout recent program rounds. The most recent analysis of 2018 post-program evaluations found themes of greater connection, modelling of wellbeing and reduced isolation. The results suggest that the peer mentor program is beneficial for individuals living with an eating disorders and meets some aims set out by the program.

### O21 What prevents young people from seeking help? Barriers towards help-seeking across at risk and clinical samples

#### Kathina Ali^1,2,3^, Daniel Fassnacht^1,2^, Louise Farrer^1,2^, Elizabeth Rieger^1,3^, Markus Moessner^4^, Kathleen Griffiths^3^, Stephanie Bauer^4^

##### ^1^Research School of Psychology, Australia; ^2^Centre for Mental Health Research; ^3^The Australian National University; ^4^Centre for Psychotherapy Research, University Hospital Heidelberg

###### **Correspondence:** Kathina Ali (kathina.ali@anu.edu.au)

Current evidence suggests that a minority of individuals with eating disorders seek and receive professional help. Several potential factors have been identified to impede help-seeking, including stigma and shame, denial of the problem, low motivation to change, negative attitudes towards seeking help, lack of knowledge about resources, and practical barriers (e.g., cost). However, there is a paucity of quantitative research examining barriers towards seeking help for eating disorders, especially among young people at risk and in clinical samples. Data was collected using an online survey among 18–25 year-old individuals in Australia. Overall, 315 participants at risk or with an eating disorder completed measures of disordered eating behaviours and attitudes, help-seeking barriers, intentions, and behaviour. The majority of participants reported denial, self-sufficiency, a fear of losing control over the illness and not wanting others to worry about their problems as the greatest barriers towards seeking help. After controlling for eating disorder symptomatology and help-seeking attitudes, stigma and shame, self-sufficiency, the fear of losing control over the illness, and not wanting others to worry uniquely predicted low help seeking from a professional source. The findings highlight the importance to educate young people about the severity of the illness and emphasise the need to seek professional help, as well as increase the awareness of help-seeking barriers among clinicians.

### O22 Universal prevention of eating disorders: Preliminary results from a 4-arm cluster randomised controlled trial evaluating teacher-led body image interventions for secondary schools

#### Melissa Atkinson^1^, Phillippa Diedrichs^2^, Jade Parnell^2^, Georgia Treneman-evans^2^

##### ^1^University of Bath, United Kingdom; ^2^University of the West of England, United Kingdom

###### **Correspondence:** Melissa Atkinson (m.j.atkinson@bath.ac.uk)

This study aimed to compare approaches to universal eating disorder prevention; specifically, three different body image interventions delivered by teachers to mixed-sex adolescents. Twenty UK schools (*N* = 2044, aged 13–15, 50.3% male) were randomised to receive one of three 5-lesson interventions (mindfulness, *n* = 514; dissonance, *n* = 541; multifaceted, *n* = 489) or classes as usual (control, *n* = 503). Self-report measures of key risk factors for eating disorders were completed at baseline, post-intervention, 6-month, and 12-month follow-up. Multilevel models assessed main and interaction effects of condition, time and gender over post-intervention and follow-up, controlling for baseline. Analysis of post-intervention data indicated no significant differences between the 4 conditions on any risk factor outcomes. Subsequent moderation analysis indicated that students with higher baseline levels of depression, anxiety, and stress reported differential benefits: favouring the multifaceted programme for improving global body esteem (*p* = 0.49), body areas satisfaction (*p* = .009), appearance internalization (*p* < .001), and life engagement (*p* = .017); and the mindfulness-based programme for improving negative affect among boys (*p* = .045). Low to moderate acceptability was reported by students in all interventions, highlighting future work to increase engagement. These findings indicate some differential intervention benefits for certain groups of students when delivered in a sustainable teacher-led format. Analysis of follow-up is required prior to firm conclusions and will be presented along with the above findings.

### O23 Media Smart-Targeted: outcomes from an Australia and New Zealand-wide pragmatic online eating disorder risk reduction trial for young adults

#### Simon Wilksch^1^, Anne O’Shea^2^, C. Barr Taylor^3^, Denise Wilfley^4^, Corrina Jacobi^5^, Tracey Wade^1^

##### ^1^Flinders University, Australia; ^2^Advanced Psychology Services; ^3^Stanford University, USA; ^4^Washington University, USA; ^5^Technische Universität Dresden, Germany

###### **Correspondence:** Simon Wilksch (simon.wilksch@flinders.edu.au)

Background: Efficacious eating disorder (ED) risk reduction programs that can be delivered at-scale are needed.

Methods: An online pragmatic, randomized-controlled trial was conducted with *N* = 575 young-women seeking to improve their body image (M age = 20.80 years). Media Smart-Targeted (MS-T) and Student Bodies (SB) were both 9-module interventions released weekly, whilst control participants received positive body image information. Primary (Eating Disorder Examination–Questionnaire [EDE-Q] Global), secondary (ED risk factors) and tertiary (ED diagnosis) measures were completed at baseline, post-program, 6- and 12-month follow-up.

Results: Primary intention-to-treat (ITT) analyses revealed no differences between groups for the EDE-Q Global, while measure completer analyses found MS-T had significantly lower scores than controls at 12-month follow-up. Secondary ITT analyses found MS-T participants reported significantly higher quality of life–mental relative to both SB and controls (6-month follow-up), while MS-T and controls had lower clinical impairment relative to SB (post-program). Amongst measure completers MS-T scored significantly lower than controls and SB on 5 variables. MS-T reduced ED onset by 66% (in those asymptomatic at baseline) and increased remission rates by 75% (in those symptomatic at baseline) relative to controls at 12-month follow-up.

Conclusions: Whilst further investigations are necessary, MS-T has fully automated procedures, low implementation costs, and the potential to be delivered at-scale to assist those assist those where face-to-face services are limited.

### O24 Promoting Confident body, Confident Child (CBCC) in the Gold Coast community: A mixed methods implementation study

#### Lyza Norton^1^, Shelley Roberts^2^, Vicki Attenborough^1^, Narelle O’Connor^1^, Laura Hart^3^, Susan Moloney^1^, Francoise Butel^1^

##### ^1^Gold Coast University Hospital, Australia; ^2^Griffith University, Australia; ^3^La Trobe University, Australia

###### **Correspondence:** Lyza Norton (lyza.norton@health.qld.gov.au)

Introduction: Body dissatisfaction is a predictor for low self-esteem, depression, unhealthy eating patterns, overweight/obesity and eating disorder development in children/adolescents. Foundations for negative body image develop in early childhood, hence prevention programs should target young children. Confident Body, Confident Child (CBCC) is an innovative, evidence-based program providing parenting strategies to promote healthy eating, physical activity and body satisfaction in children aged 2–6 years. This study aims to evaluate the population-level implementation of CBCC by training Child Health Nurses (CHNs) across Gold Coast Health (GCH) to disseminate CBCC to parents of young children in their usual clinics.

Method and Participants: This study uses an implementation-effectiveness hybrid design, with dual focus on assessing implementation (process evaluation) and clinical effectiveness (outcomes evaluation). CBCC implementation will be done at two levels: CHN training and parent delivery. Process data on CBCC reach, dose and fidelity among CHNs and parents and outcome data on changes in CHN and parent knowledge, attitudes and behaviours pre- and post-intervention will be collected. CHN and parent acceptability will also be assessed through interviews.

Results: Preliminary data on implementation processes will be available for the conference.

Conclusion: This study involves optimising existing community health services and addressing an identified gap in current care. This is the first study to evaluate population-wide CBCC implementation in a real-world health service setting.

### O25 Extending body image intervention from the classroom to the home: an evaluation of raising confident girls: A school-based body image intervention for mothers, delivered alongside a classroom-based body image intervention for daughters at an independent age

#### Jody Forbes^1^, Zali Yager^2^, Susan Paxton^3^

##### ^1^Brisbane Girls Grammar School, Australia; ^2^Victoria University, Australia; ^3^La Trobe University, Australia

###### **Correspondence:** Jody Forbes (jforbes@bggs.qld.edu.au)

This presentation describes the evaluation of a school-based body image intervention, Raising Confident Girls (RCG), developed for Year 8 mothers at an independent girl’s school. The 3-session seminar was delivered by the School Psychologist to a group of 60 mothers. Alongside RCG, Year 8 daughters participated in the classroom-based body image program delivered by teachers.

RCG incorporates etiological theory and focuses on reducing known causal risk factors for body dissatisfaction including internalization of thin-ideal, body comparison and appearance focused conversations. Incorporating a number of research based resources within the body image field, the seminar was educative, experiential, interactive and multi-sessional. The first session, Embrace, included a viewing of the Embrace documentary. During the second session, Educate, participants discussed the risk factors for body dissatisfaction. Empower, the final session, focused on understanding adolescent development and using mother-daughter relationships to encourage positive body image. Each session, participants were provided with booklets and were required to participate in homework exercises from the Body Project.

Multilevel-modelling analyses revealed compared to a control group, participants reported significant improvements in body esteem, body appreciation, parenting knowledge, positive role modelling, parenting skills and confidence at post-test and 3 month follow-up. Findings contribute knowledge to the body-image research field and assist schools to understand how to enhance the effectiveness of classroom-based body image interventions via parental involvement.

### O26 Effects of short-term exposure to appearance-focussed thin-ideal instagram images in young women and the effectiveness of a brief social media literacy intervention

#### Brigitte Caruana^1^, Eleanor Wertheim^1^, Fay Anixiadis^1^, Rachel Rogers^2^

##### ^1^La Trobe University, Australia; ^2^College of Social Sciences and Humanities, Northeastern University, Boston, MA, USA

###### **Correspondence:** Brigitte Caruana (becaruana@students.latrobe.edu.au)

Recent research has raised concerns about the influence of social media on body dissatisfaction in young women. This study examined the effect of exposure to objectified appearance-focused Instagram-like images on state mood and body dissatisfaction in young women, and tested the effectiveness of a short, theoretically designed social media literacy (SML) intervention. Female participants (*N* = 144) completed trait measures of potential moderators and state body dissatisfaction and mood scales, and were randomly assigned to the SML intervention followed by exposure to objectified female images or to an Instagram general-information video followed by those objectified images or non-appearance-focused control images. Results showed exposure to the objectified images resulted in greater increases in body dissatisfaction and negative mood than exposure to control images. Furthermore, from pre-intervention to post-image-exposure the intervention group reported significantly fewer body dissatisfaction increases than the objectified condition; however, this was not the case when looking at changes from immediate post-intervention to post-image-exposure. Findings suggest that while the intervention was generally received well and ameliorated body dissatisfaction and negative mood in the moment, it did not protect against subsequent exposure to multiple objectifying images. Thus, exposure to appearance focussed Instagram-like images may increase body dissatisfaction and negative mood in women and social media literacy may play a limited role in protecting against these apparent negative effects.

### O27 Developing an ethical psychological assessment tool for use in the cosmetic industry

#### Toni Pikoos^1^, Simone Buzwell^1^, Nektaria Tzimas^2^

##### ^1^Swinburne University of Technology, Australia; ^2^CPD Institute, Australia

###### **Correspondence:** Toni Pikoos (tpikoos@swin.edu.au)

The number of people seeking appearance-altering cosmetic treatments increases rapidly each year. A number of psychological factors have been associated with poorer outcomes from cosmetic treatments, such as a history of depression and anxiety, relationship concerns and the presence of Body Dysmorphic Disorder (BDD). Cosmetic treatments have been noted to worsen BDD symptoms, as the results often fail to meet their expectations, leading to further preoccupation with appearance and cosmetic treatment seeking. The current project aimed to develop and pilot an assessment tool which could detect individuals with psychological contraindications for cosmetic treatment. The screening tool was piloted with 104 individuals seeking minimally invasive cosmetic treatments, such as Botox and dermal fillers. Results revealed several factors which predicted heightened and unrealistic expectations for cosmetic treatments, which in turn led to reduced satisfaction with treatment outcome. These included elevated BDD symptoms, high levels of depression and anxiety and younger age. Results emphasised that younger people may seek aesthetic treatments as a means to build self-esteem or improve psychological symptoms. The current research provided further evidence that psychological screening is necessary within cosmetic treatment settings and can help guide a more ethical informed consent process. Screening results may provide a basis for cosmetic practitioners to refer at-risk clients on to mental health professionals for further assessment or to receive evidence-based treatments.

### O28 The longitudinal and reciprocal relationships between selfie activities and self-objectification and appearance concerns among adolescents

#### Yuhui Wang^1^, Xiaochun Xie^2^, Jasmine Fardouly^3^, Lenny Vartanian^4^, Li Lei^1^

##### ^1^Renmin University of China; ^2^Northeast Normal University, China; ^3^Macquarie University; ^4^UNSW Australia

###### **Correspondence:** Yuhui Wang (yuhui2016@ruc.edu.cn)

Although a few studies have examined the cross-sectional associations between selfie activities (e.g., selfie-posting and selfie-editing) and self-objectification or body image, little is known about whether bidirectional relationships exist between selfie activities and these body-related variables over time. The present study examined the reciprocal relationships between selfie activities (i.e., selfie-posting, selfie-editing, and selfie-viewing) and both self-objectification and appearance concerns (i.e., body dissatisfaction and facial dissatisfaction) among adolescents using a longitudinal design. Chinese adolescent boys and girls (*N* = 767) completed questionnaires of the key constructs at baseline and at 6-month follow-up. These measures included: selfie-posting, selfie-editing, selfie-viewing, self-objectification, facial dissatisfaction, and body dissatisfaction. Results indicated that selfie-editing and selfie-viewing, but not selfie-posting, generally predicted increases in adolescents’ self-objectification and appearance concerns overtime. Adolescents’ antecedent levels of self-objectification contributed to increases in their subsequent selfie activities. In addition, adolescents’ facial dissatisfaction predicted selfie-viewing and selfie-editing but not selfie-posting, whereas body dissatisfaction had no influence on selfie activities among adolescents. Findings from the current study provide new insights into the reciprocal relationships between selfie activities and body image.

### O29 Do as I do: Maternal role modelling of positive body image in a large community sample

#### Stephanie R. Damiano^1^, Zali Yager^2^, Ivanka Prichard^3^, Laura Hart^1^

##### ^1^La Trobe University, Australia; ^2^Victoria University, Australia; ^3^Flinders University, Australia

###### **Correspondence:** Stephanie R. Damiano (stephanie.damiano@gmail.com)

Mothers are important role models of body image attitudes and behaviours for their children. Little is known about maternal self-perceptions of such modelling and the characteristics of mothers who are engaging in role modelling of positive body image. The aim of this study was to examine the relationships between role modelling and other maternal attitudes and behaviours, and to identify mothers who may require more assistance in being a positive body image role model for their children. Participants were 887 mothers (M age = 43.98 years) who completed an online questionnaire assessing their level of modelling of positive body image attitudes and behaviours, body appreciation, and dietary restraint. More positive role modelling was associated with greater body appreciation and lower levels of dietary restraint. Mothers in the higher weight categories reported less positive role modelling than mothers in the middle weight category. Mothers of younger children (aged between birth and 10 years) reported more positive role modelling than mothers of adolescent and adult children. These findings have important implications for the development of targeted interventions for mothers to improve maternal body attitudes and behaviours and promote positive behaviours and outcomes for children.

### O30 Do the levels of body esteem and dietary restraint remain stable in girls from age 6 to age 8?

#### Stephanie R. Damiano^1^, Siân McLean^2^, Susan Paxton^1^

##### ^1^La Trobe University, Australia; ^2^Victoria University, Australia

###### **Correspondence:** Stephanie R. Damiano (stephanie.damiano@gmail.com)

Tracking risk factors for eating disorders from early childhood is important as increasing evidence demonstrates that unhealthy attitudes and behaviours develop from a young age. The aim of this study was to examine stability and change in girls’ body esteem and dietary restraint from age six to eight. As part of The Children’s Body Image Development Study, 107 girls participated in annual interviews at ages six, seven, and eight, which included assessment of body esteem and dietary restraint. We found that girls who reported low levels of body esteem at age six continued to report significantly lower levels of body esteem at ages seven and eight than girls with initial moderate and high levels of body esteem. Similarly, girls who reported high levels of dietary restraint at age six reported significantly higher levels of dietary restraint at ages seven and eight compared with girls with low dietary restraint at six. Analyses revealed medium to large effect sizes. These findings indicate that body image and dieting behaviours may be stable over this three year period and highlight that a small proportion of young girls may be vulnerable to experiencing ongoing concerns from a young age. This has important implications for eating disorder prevention and suggests that intervention, with parents or delivered through schools, may be appropriate from early childhood.

### O31 A qualitative exploration of the relationship between body image and exercise for mothers of children aged 0–5 years

#### Anita Raspovic^1^, Laura Hart^1^, Zali Yager^2^, Ivanka Prichard^3^

##### ^1^La Trobe University, Australia; ^2^Victoria University, Australia; ^3^Flinders University, Australia

###### **Correspondence:** Anita Raspovic (a.raspovic@latrobe.edu.au)

The relationship between body image and exercise in early motherhood may significantly influence mother and infant wellbeing. Using a qualitative, history-based approach, mothers’ lived experiences of the relationship between body image and exercise were explored. Semi-structured interviews with 21 mothers (0–5 years postpartum), were conducted using a schedule designed to elicit narratives about body image in the postpartum period and any impact on exercise patterns. Verbatim transcripts were examined using thematic and content analysis (Braun & Clarke, 2013) to establish body image profiles (i.e., relative levels of dissatisfaction and appreciation), and then to examine mothers’ exercise patterns and perceived relationships with body image. Twenty percent of transcripts were double-coded to enhance coding reliability. Results demonstrated that women’s experiences with body image and exercise were individualistic, dynamic and complex. Body image and exercise were enhanced postpartum in some mothers, but diminished in others, with five discrete body image/exercise profiles apparent:1) average BI/average exercise, 2) dissatisfied BI/low exercise, 3) dissatisfied BI/high exercise, 4) appreciative BI/health motivated exercise, and 5) appreciative BI/weight management exercise. Dissatisfied profiles posed greatest risk to mothers’ wellbeing, due to body-related distress and aberrant exercise. Conversely, appreciative profiles were associated with more adaptive exercise behaviours. Findings suggest that reducing body dissatisfaction and improving body appreciation in early motherhood may assist in encouraging healthy exercise at this important time.

### O32 Associations between parental teasing in regards to their child’s weight, body and/or shape, and eating problems in adolescents: a systematic review

#### Phillipa Hay^1^, Lucy Dahill^1^, Stephen Touyz^2^, Natalie Morrison^3^

##### ^1^School of Medicine and Centre for Health Research Western Sydney University, Australia; ^2^University of Sydney, Australia; ^3^Translational Health Research Institute, School of Medicine, Western Sydney University, Australia

###### **Correspondence:** Lucy Dahill (l.dahill@westernsydney.edu.au)

Objective: To investigate associations between adolescent eating problems and exposure to parental teasing regarding child weight, body, and or shape.

Method: A systematic search of the literature (using PubMed, Ovid Medline, CINAHL, SCOPUS, PsychINFO, EMBASE, Cochrane and Web of Science) was undertaken to identify relevant literature published in English or French, related to adolescents aged 10–19 years, and published between the period January 1980 to October 2018.

Results: 13 studies met criteria for inclusion. The studies were predominantly cross-sectional (*n* = 9) including one thesis paper and three longitudinal studies. Although parents teasing was most often less frequent than that from peers and siblings, the impact of parental teasing was significant and also impacted on sibling teasing. Three key themes for associations emerged: Parents as Socialisers of Adolescents’ Appearance Concerns, Emotional Internalization, and Family Environment.

Discussion: There is evidence to support an association between eating problems in adolescents and exposure to parental teasing in regards to their child’s weight, body or shape. Parents may be unaware of the impact of the words they use or the wider influence these words may have. Future research should address current gaps in the literature by exploring adolescents’ perceptions of parents’ positive and negative words around body image in longitudinal studies with representative.

### O33 Developing consensus on Australian eating disorder research priorities: A Delphi study

#### Laura Hart^1^, Tracey Wade^2^

##### ^1^La Trobe University, Australia; ^2^Flinders University, Australia

###### **Correspondence:** Laura Hart (l.hart@latrobe.edu.au)

Purpose: To develop consensus on the priorities for funding eating disorder research in Australia a Delphi study was conducted.

Methods: An electronic survey presenting 29 research areas grouped under 7 domains (Accessible Evidence Based Treatments, Origins of Eating Disorders, Early Detection and Early Intervention, Prevention, Social and Emotional Determinants in Eating Disorders, Comorbidity and Suicidality, Under-served and Under-researched Groups) was sent to all Australian members of Australia and New Zealand Academy for Eating Disorders and the National Eating Disorders Collaboration.

Results: Participants (*N* = 291, aged 18–68, 92% female) were assigned to one of three ‘panels’ based on their expertise; Eating Disorder Specialists (*n* = 89, 45%), Consumer/Carers (*n* = 77, 38%) and Affiliates (*n* = 35, 17%). Research areas rated as ‘Essential’ or ‘Important’ by more than 80% of all three panels were endorsed as a priority. Near miss items were entered into a second round for re-rating. Accessible Evidence Based Treatments was considered the top priority, followed Early Detection and Early Intervention, then Prevention. Research areas describing treatments and interventions were consistently endorsed across all panels, with areas examining aetiology or comorbidity less well received.

Conclusions: The Delphi expert consensus method is a useful tool for developing research funding priorities in the eating disorders field across different groups and the outcomes of this study can be usefully employed by Australian funding bodies.

### O34 Anxiety and disordered eating: A common genetic pathway influencing symptoms of adolescent females?

#### A. Kate Fairweather-Schmidt, Tracey Wade

##### Flinders University, Australia

###### **Correspondence:** A. Kate Fairweather-Schmidt (kate.fairweather-schmidt@flinders.edu.au1)

Objective: Numerous clinical observations note the co-occurrence of anxiety and disordered eating, where an abundance of papers have employed a variety of methodologies to examine this overlap. To date, investigations into the potential of a genetic liability for both conditions continue to be limited by design or sample.

Methods: Teenage female twins aged 12–15 and 16–19 years (Wave 1: *N* = 699, 351 pairs; Wave 2: *N* = 669, 338 pairs) were interviewed by telephone using the Eating Disorder Examination (EDE), and also completed measures related to disordered eating risk, including Children’s Anxiety Sensitivity Index (CASI).

Results: A multivariate common pathway model with inclusion of additive genetic (A) and non-shared environment (E) influences fit data most parsimoniously. A general anxiety sensitivity factor was predominately heritable (A = 0.75; 95% CI = 0.54–0.88); the remaining variance was attributable to non-shared environment influences (44.10%). The CASI latent factor contributed equivalent, but non-significant influences on phenotypic CASI (0.57) and EDE (0.58). Multinomial logistic regression indicated that weight-related peer teasing (RRR = 2.53; 95% CI = 1.09–5.86) was able to differentiate those with high CASI from high EDE scores.

Conclusion: Liability to anxiety sensitivity is characterised by genetic and non-shared environmental influence, possessing signals of covariation between anxiety sensitivity and disordered eating. Genetic analysis suggests that a single latent CASI factor influences both CASI and EDE phenotypes.

### O35 Stigma towards bulimia nervosa in Australia and China

#### Daniel Fassnacht, Rodney Cassel

##### The Australian National University, Australia

###### **Correspondence:** Daniel Fassnacht (daniel.fassnacht@anu.edu.au)

There is a lack of cross-cultural studies examining stigmatising attitudes towards eating disorders, particularly towards bulimia nervosa. The aim of the current study was to investigate stigma towards people with bulimia nervosa compared to depression and type 2 diabetes in Australia and China. Further, the level of contact and knowledge about the illness as well as other theoretical explanations as potential correlates of stigma were explored. Overall, 430 participants across Australia and China were randomly assigned to a vignette describing either a fictive character with bulimia nervosa, depression or type 2 diabetes; then their level of stigma was assessed. Results showed that Chinese in comparison to Australian participants had the lowest level of knowledge and highest level of stigma towards all conditions. Further, the characters depicting bulimia nervosa and type 2 diabetes received greater blame-related stigma, whereas, the character with depression was more distrusted and participants reported a greater desire for social distance. The current findings provide insight into the types and levels of stigma towards bulimia nervosa in Australia and China. Implications and future research questions are discussed.

### O36 Examining the evidence base for Specialist Supportive Clinical Management for Anorexia Nervosa

#### Virginia McIntosh (gini.mcintosh@canterbury.ac.nz)

##### University of Canterbury, New Zealand

Specialist Supportive Clinical Management (SSCM) began as an atheoretical comparison treatment for more theory-based specialised psychotherapies. After the surprising early result that SSCM was equal to or more effective than the specialised therapies, several subsequent RCTs went on to compare SSCM with other treatments. This paper reviews the research to date for this modest treatment, describes its key elements and discusses its role in the treatment of anorexia nervosa.

### O37 Family Based Treatment for Anorexia Nervosa (FBT-AN) telehealth pilot: outcomes

#### Tania Withington^1^, Penny Knight^1^, Chanelle Sutherlane^1^, Judith Burton^2^, Esben Strodl^2^, Ros Darracott^2^, Danielle Davidson^2^

##### ^1^Children Health Queensland, Child and Youth Mental Health Service, Eating Disorder Program, Australia; ^2^Queensland University of Technology, Australia

###### **Correspondence:** Tania Withington (tania.withington@health.qld.gov.au)

FBT-AN is internationally recognised as the evidenced-based first-line treatment for adolescents diagnosed with anorexia nervosa. Access to FBT-AN trained clinicians for families living outside major metropolitan areas is limited particularly in large geographically diverse states such as Queensland Australia. To address this inequity of access to evidenced-based treatment options, the Children’s Health Queensland, Child and Youth Mental Health Service, Eating Disorder Program invested in a 2-year pilot project offering FBT-AN to families across Queensland. Families with a child or adolescent diagnosed with Anorexia Nervosa, who met criteria for FBT-AN, and were engaged with their local CYMHS service, were offered the opportunity to participate in the study investigating the efficiency and effectiveness of FBT-AN using video-conferencing as the medium of service delivery. A total of 28 families were offered a place in the pilot project, including 5 families seen face-to-face for comparison purposes. This paper will present an overview of the outcomes of this study measured using standardised assessment tools and quantitative data analysis. Implications for future treatment service delivery will be discussed.

### O38 Collaborative meetings with young people with eating disorders and their families

#### Rachel Barbara-May (r.barbara-may@alfred.org.au)

##### Alfred Child and Youth Mental Health Service, Australia

The Eating Disorders Program at the Alfred Child and Youth Mental Health Service (CYMHS) provides integrated, multi-disciplinary outpatient treatment to children and young people affected by eating disorders and their families. The program aims to provide intervention at the earliest possible time to promote good outcomes for young people. As the program is integrated within a child and youth mental health service, young people and families have access to a full suite of mental health services, including case management and specialist interventions as required. The program operates from key principles of ‘recovery is expected’, ‘families are engaged in a purposeful partnership’ and ‘parents are the best resource for change’. This presentation will provide a description of the new Family Brief Intervention (FBI) that has been designed specifically for those young people presenting with early concerns around food and eating. Drawing on single session ideas, this is a collaborative family therapy approach that fully integrates and utilises multi-disciplinary and lived experience expertise. The presentation will discuss the pre-meeting process and set up, the in-meeting clinical process and tailored follow up and continuing care for families and young people. Outcome results will be shared and there will be a reflection from the family peer worker involved in the meetings.

### O39 Changes in serum thiamine levels during nutritional rehabilitation of adolescent patients hospitalised with anorexia nervosa

#### Elizabeth Parker^1^, Terri Maister^2^, Anita Stefoska-Needham^2^, Christine Wearne^1^, Gail Anderson^1^, Linette Gomes^1^, Simon Clarke^1^, Michael Kohn^1^

##### ^1^Westmead Hospital, Australia; ^2^University of Wollongong, Australia

###### **Correspondence:** Elizabeth Parker (Elizabeth.Parker@health.nsw.gov.au)

Aim: To investigate whether adolescent patients hospitalised with anorexia nervosa (AN) provided with nutritional rehabilitation void of thiamine supplementation, develop thiamine deficiency during their admission.

Methods: A retrospective clinical audit was conducted of patients with AN receiving nutritional rehabilitation on an adolescent ward in a Western Sydney Hospital, from 2016 to 2017. A total of 167 admissions were reviewed, of which 60 had serum thiamine levels measured. Data was extracted from paper-based and electronic medical records, including serum thiamine levels and supplementation history. A linear mixed effects model was employed for analysis.

Results: During hospital admission, median thiamine levels increased by 9.2 nmol/L per week (*p* < 0.001). No patients developed thiamine deficiency, however one patient was admitted with serum thiamine levels below the normal range at 62 nmol (normal range 67 – 200 nmol/L) which resolved by the second week of admission. In 15 admissions (25%), serum thiamine levels were observed to be above the normal reference range. No patients received thiamine supplementation during their admission, with oral diet and/or enteral feeds and a daily multivitamin being their main source of thiamine.

Conclusions: Nutritional management of adolescents hospitalised with AN can be safely commenced without separate thiamine supplementation. The results of this study suggest a daily multivitamin in addition to the thiamine provided by oral dietary intake, with or without enteral feeds, is adequate to meet adolescents’ requirements.

### O40 Piloting a new approach to ARFID treatment

#### Danielle Pogos^1,3^, Elizabeth K Hughes^1,2,3^, Susan M Sawyer^1,2,3^, Erica Allan^1^, Claire Burton^1,3^

##### ^1^Royal Children’s Hospital, Australia; ^2^University of Melbourne, Australia; ^3^Murdoch Children’s Research Institute, Australia

###### **Correspondence:** Danielle Pogos (danielle.pogos@rch.org.au)

There is currently no evidence-based treatment for Avoidant/Restrictive Food Intake Disorder (ARFID) due to its recent specification in the DSM-5. In the absence of known efficacious treatments for ARFID, services face the challenge of adapting existing treatments for other disorders. Two examples of relevant treatments are Family Based Treatment (FBT) due to its effectiveness in promoting weight gain in individuals with Anorexia Nervosa, and the Unified Protocols (UP), a flexible, cognitive-behavioural treatment that is effective in treating a range of psychological disorders. These two approaches have been integrated into one treatment protocol, FBT + UP, by the Centre for the Treatment of Eating Disorders in Minnesota. The Royal Children’s Hospital Eating Disorders Service is one of several international sites piloting and evaluating this treatment in order to assess its feasibility and acceptability in preparation for an eventual multi-centre randomised controlled trial. It has been challenging to assess and treat a heterogeneous group of patients experiencing a newly defined disorder, given the paucity of evidence to guide practice. Diagnostic challenges include assessment of clinically significant body image concerns, while treatment challenges include the transition from a family based approach to individual treatment, patient engagement, and adapting the treatment to suit varied patient groups. This presentation will discuss the implementation experience of the FBT + UP in the context of these new challenges.

### O41 Service innovations in intensive treatment in a regional/rural public health service

#### Vyv Rodnight^1^, Beth Shelton^2^, Rachel Knight^3^, Michelle Roberton^3^

##### ^1^La Trobe University, Australia; ^2^NEDC, Australia; ^3^Victorian Centre of Excellence in Eating Disorders, Australia

###### **Correspondence:** Vyv Rodnight (v.rodnight@latrobe.edu.au)

Multi-family therapy for anorexia nervosa (MFT-AN) is a relatively new intensive treatment with an emerging evidence base. Bendigo Health Child and Adolescent Mental Health Service (BH-CAMHS) is a public mental health service that has provided evidence-based, family-centred, parent-led re-feeding interventions for adolescent anorexia nervosa as core business over the last seven years to regional and rural communities. The Victorian Centre of Excellence in Eating Disorders (CEED) is a state-wide service providing secondary case consultation, professional development and service development support in eating disorders to the Victorian Specialist Mental Health System. Over the last four years CEED clinicians have trained in and delivered MFT-AN, and collaborated with public health services on MFT-AN delivery. Over the last two years Bendigo BH-CAMHS and CEED have collaborated to pilot and embed MFT-AN as an innovative treatment intervention that augments single Family-Based Treatment. The collaboration has enhanced family and clinician confidence and empowerment, and lead to improved treatment outcomes for rural families experiencing anorexia nervosa. This presentation will outline and discuss the service collaboration between BH-CAMHS and CEED, explore piloting and embedding the program at BH-CAMHS and provide qualitative acceptability and impact data from families, acceptability data from clinicians and service leaders and indicative outcome data.

### O42 Personality disorders and eating disorders: A review of past and more recent research and the implications for understanding eating disorder treatment

#### Tanya Gilmartin (tanyalgilmartin@gmail.com)

##### RO-DBT Australia

Research over several decades has indicated high comorbidity between eating disorders and personality disorders. Although a search through the research literature yields a range of results, a general implication that can be made is that there is an association between Bulimia nervosa and cluster B personality disorders, particularly Borderline Personality Disorder, and an association between anorexia nervosa and cluster C personality disorders, particularly Obsessive Compulsive Personality Disorder. As an individual’s personality is somewhat stable throughout that person’s lifespan, it can be implied that targeting a person’s underlying personality pathology may assist in improving treatment outcomes for that same person’s eating pathology. With the publication of the DSM-V, an alternate model of personality disorder diagnoses was proposed. This model was based on dimensional approaches to personality rather than categorical diagnoses. This presentation will review previous research on categorical personality disorder diagnoses and the comorbidity with eating disorders, as well as outline emerging research findings regarding dimensional models of personality disorders and eating disorder comorbidity. The presentation will also outline how past and emerging research can assist clinicians in understanding, selecting and developing treatment opportunities for eating disorders.

### O43 The role of early maladaptive schemas in eating disorders

#### Jinyuan Queenie Wu, Ross king

##### Deakin University, Australia

###### **Correspondence:** Jinyuan Queenie Wu (Wjiny@deakin.edu.au)

Those with EDs show a high level of co-morbidity of both medical and psychiatric illnesses, and many display particular complex personality traits. Given that existing treatments for EDs are not successful with all individuals, it is important to consider potential underpinning factors so as to guide the development of new treatment approaches. Early maladaptive schemas (EMSs) are suggested to be associated with complex psychopathology and in the past decade, Schema theory has gained significant interest from researchers focused on prevention and treatment approaches for EDs. Our study compared directly the level of EMSs in 121 ED females via an online survey. They were divided into 4 subtypes of ED according to participants’ self-reported diagnosis and examination of BMI and reported ED behaviours according to DSM-5 criteria: 13 AN-R type, 60 AN-BP type, 42 BN type, and 6 BED type (BED). Eating Disorder Examination Questionnaire (EDEQ-6.0) and Young Schema Questionnaire (YSQ-S3) were used. Despite limitations, our findings suggested that certain patterns of EMSs were associated with ED pathologies and behaviours, which differed between diagnostic groups.The AN-R group reported less eating concern and weight concern than BN group, and AN-BP group reported less eating restraint than BED group.Clinical significance of this study helps to understand EDs further, and support further studies examining the potential use of schema therapy in EDs.

### O44 Attention to detail, cognitive rigidity, and disordered eating: The mediating role of difficulties identifying feelings

#### Sarah Giles^1^, Elizabeth Hughes^1,2,3^, Matthew Fuller-Tyszkiewicz^4^, Isabel Krug^1^

##### ^1^The University of Melbourne, Australia; ^2^Murdoch Children’s Research Institute, Australia; ^3^Royal Children’s Hospital, Australia; ^4^Deakin University, Australia

###### **Correspondence:** Sarah Giles (giless1@student.unimelb.edu.au)

Objective: The cognitive-interpersonal model proposes that obsessive-compulsive traits characterized by high levels of attention to detail and cognitive rigidity confer risk for the development of eating disorders (ED). While socio-emotional deficits such as alexithymia, contribute to its maintenance. We aimed to investigate the mediating role of specific alexithymia traits (difficulties describing feelings, difficulties identifying feelings, and externally orientated thinking) on the relationship between attention to detail, cognitive rigidity, and ED symptoms, while controlling for anxiety and depression symptoms.

Method: Four-hundred-and-one female university students (M = 20.57, SD = 4.99 years-old) completed several self-report questionnaires including; Detail and Flexibility Questionnaire (DFlex); Toronto Alexithymia Scale (TAS); Hospital Anxiety and Depression Scale; and the Eating Disorder Examination Questionnaire.

Results: Path-analyses revealed a significant direct effect from DFlex-Cognitive rigidity subscale to ED symptoms, but no significant direct effect was observed between DFlex-Attention to detail subscale and ED symptoms. The TAS-Difficulties identifying feelings subscale was a significant mediator of the relationship between attention to detail and ED symptoms and between cognitive rigidity and ED symptoms. However, after controlling for anxiety and depression only the cognitive rigidity to ED symptoms path remained significant.

Conclusions: Difficulties identifying feelings may in part underlie the relationship between attention to detail and cognitive rigidity and ED symptoms, but this relationship is highly influenced by co-morbid anxiety and depression symptoms.

### O45 The contribution of intolerance of uncertainty to psychopathology, eating-related anxiety, ritualised behaviour and cognitive rigidity in a clinical sample with anorexia nervosa: Findings from a longitudinal study

#### Alice Kesby^1^, Jessica Grisham^1^, Sarah Maguire^2^, Janice Russell^3^

##### ^1^UNSW, Sydney; ^2^InsideOut Institute, University of Sydney; ^3^RPAH, Northside Clinic, University of Sydney

###### **Correspondence:** Alice Kesby (a.kesby@psy.unsw.edu.au)

Evidence supports Intolerance of Uncertainty (IU) as transdiagnostic mechanism across a range of psychiatric disorders, particularly in populations where anxiety symptoms predominate. Fear and anxiety regarding food, eating, weight and body shape are at the core of Anorexia Nervosa (AN), yet the cognitive processes underlying anxiety and anxiety-driven behaviour remain poorly understood and insufficiently targeted in this illness group. Literature supports heightened IU in AN populations, and has shown that these individuals exhibit a particular susceptibility to experience distress in response to experimental conditions of uncertainty. Ritualistic eating behaviour and extreme dietary restriction may represent excessive approach and avoidance responses that develop as a means of coping with poor uncertainty tolerance. This study aimed to examine whether IU represents a potential mechanism underlying anxiety, ritualistic behaviours and cognitive inflexibility in a clinical sample (*N* = 50) undergoing treatment for AN. A repeated measures longitudinal design was used to examine the contribution of trait IU to core eating disorder symptoms, ritualised behaviour, cognitive flexibility and treatment outcomes following four weeks of inpatient or day program treatment. Further, an in vivo consumption paradigm was used to investigate whether state IU contributed to patient anxiety at three time-points (baseline, pre-eating, post-eating) relative to eating an energy-dense meal. Preliminary findings are presented and clinical implications regarding IU as a possible adjunctive treatment target are discussed.

### O46 Photo-editing: A stronger predictor of multidimensional body image concerns in young men than women

#### Siân McLean^1^, Lucy Chen^1^, Susan Paxton^2^, Amy Slater^3^

##### ^1^Victoria University, Australia; ^2^La Trobe University, Australia; ^3^The University of the West of England, Australia

###### **Correspondence:** Siân McLean (sian.mclean@vu.edu.au)

Background: Engagement with appearance-related activities on social media, including editing photos for improving self-presentation, is associated with higher body image and eating concerns. However, few studies have examined photo-editing in men. This study aimed to address this gap and explore relationships between gender, social media use, and photo-editing, and multidimensional constructs of body image: appearance satisfaction, appearance investment, and fear of negative appearance evaluation amongst young adults.

Method: Men (*n* = 188) and women (*n* = 182) aged 18–25 years completed self-report measures.

Results: Women used social media and engaged in photo-editing significantly more frequently than men (d = .63; d = .87, respectively). Regression analyses revealed that gender and social media use were significant predictors of fear of negative appearance evaluation and appearance investment, respectively. Photo editing and the gender-by-photo-editing interaction were significant predictors of both appearance investment and fear of negative appearance evaluation. The interaction indicated that the associations between photo editing and both body image variables were stronger for men than for women. Appearance satisfaction was not associated with independent variables.

Conclusion: Findings suggest that photo-editing may be an appropriate target for body image interventions for both men and women as these activities can promote self-scrutiny that may lead to or maintain body image concerns. Further research is required to determine the temporal direction of these relationships.

### O47 “I wish I was as skinny as her”: The roles of appearance comparisons, body-related thoughts and critical media awareness in response to exposure to thin-ideal Instagram images

#### Fay Anixiadis^1^, Eleanor Wertheim^1^, Brigitte Caruana^1^, Rachel Rogers^2^

##### ^1^La Trobe University Melbourne, Australia; ^2^College of Social Sciences and Humanities, Northeastern University, Boston, MA, USA

###### **Correspondence:** Fay Anixiadis (fay.an@hotmail.com)

Research to date has suggested that exposure to thin-idealised images on social networking sites may contribute to young women’s body dissatisfaction and negative mood. This study aimed to examine whether exposure to Instagram-like thin-idealised images would lead to a worsening of state mood and body dissatisfaction. In an advance on prior research, women reported their thoughts while viewing these images and the extent to which the thoughts were protective or risk-promoting was examined. Female participants (*N* = 126) completed trait body dissatisfaction and body comparison measures, and state body dissatisfaction and mood visual analogue scales, and were randomly assigned to view, and report their thoughts about, either thin-idealised female images or control images containing primarily scenery. The control sample increased negative mood and decreased body dissatisfaction more than thin-ideal participants following image exposure, with a marginal interaction effect suggesting that trait appearance comparison and internalization of the thin ideal played small moderating roles as risk factors. In an extended sample that reported thoughts while viewing the thin-ideal images (*N* = 91), the frequency of upward appearance comparison thoughts and positive body-related thoughts were associated with negative mood changes, but media literacy thoughts were not. Findings are discussed in relation to potential risk and protective factors for responses to exposure to thin-idealised Instagram images, highlighting the specific methods used in this study.

### O48 Psychological distress and disordered eating in adolescents: the moderating role of emotion dysregulation

#### Nora Trompeter^1^, Kay Bussey^1^, Jonathan Mond^2^, Phillipa Hay^3^, Deborah Mitchison^1,3^

##### ^1^Centre for Emotional Health, Department of Psychology, Macquarie University, Australia; ^2^Centre for Rural Health, University of Tasmania; ^3^Translational Health Research Institute, School of Medicine, Western Sydney University

###### **Correspondence:** Nora Trompeter (nora.trompeter@mq.edu.au)

Background: Psychological distress has been recognised as a key risk factor in eating disorder psychopathology (Stice, 2002), however not all adolescents experiencing distress develop disordered eating and possible moderating factors need to be investigated. The aim of this study was to examine if emotion dysregulation moderates the relationship between psychological distress and disordered eating.

Method: The study used cross-sectional data from Wave 2 of the EveryBODY study, a large longitudinal project investigating eating disorders among Australian adolescents. Data from 2784 adolescents aged between 11 and 19 years (M = 14 years, 9 months) were used. Participants completed a self-report questionnaire about current eating pathology (EDE-Q), psychological distress (K10), emotion dysregulation (DERS-SF), and demographics.

Results: Regression analyses revealed that emotion dysregulation significantly moderated the relationship between psychological distress and fasting as well as purging, whereby the associations were stronger as emotion dysregulation increased. However, there was no significant moderating effect of emotion dysregulation for either driven exercise or binge eating.

Conclusion: These results suggest that emotionally dysregulated adolescents may be more inclined to engage in extreme weight loss behaviours including fasting and purging, behaviours commonly associated with anorexia nervosa and bulimia nervosa. Interestingly there was no moderating effect of emotion dysregulation on the relationship between distress and binge eating, despite theories proposing that this behaviour is highly related to emotion regulation.

### O49 Patterns of body image class according to valence may differentiate exercise behaviours in early motherhood. A latent profile analysis

#### Anita Raspovic^1^, Laura Hart^1^, Zali Yager^2^, Ivanka Prichard^3^

##### ^1^La Trobe University, Australia; ^2^Victoria university, Australia; ^3^Flinders University, Australia

###### **Correspondence:** Anita Raspovic (a.raspovic@latrobe.edu.au)

Pregnancy and the postpartum period are times of significant changes to women’s bodies and body image. This study conducted Latent Profile Analysis to examine whether discrete groups of body image occur postpartum (i.e., Body Image Classes; BIC), according to relative levels of body dissatisfaction and appreciation in early motherhood. BICs were subsequently examined for associations with exercise. Data from 263 mothers 0–5 years postpartum (M age = 35, SD = 5) were gathered via a previous online survey. Demographic and anthropometric data, body appreciation, dissatisfaction, and duration of weekly exercise were analysed. Fit indices for a series of two to five class latent profile models showed a three-class model as most parsimonious. The majority of the sample fell into the BIC characterised by average levels of body dissatisfaction and body appreciation (*n* = 144, 54.75%), followed by a BIC with levels of higher body dissatisfaction and lower body appreciation (*n* = 60, 22.81%). A third class showed levels of higher body appreciation and lower body dissatisfaction (*n* = 59, 22.43%). After accounting for age and body mass index there was a trend towards higher levels of exercise in the appreciative followed by the average BICs, compared to the dissatisfied BIC. This research has important implications for our understanding of positive and negative body image and the link between these constructs and the promotion of exercise in early motherhood.

### O50 Disordered eating and health-related quality of life in young adults: examining effect modification by gender

#### Laura Hart^1^, Allegra Gordon^2^, Vishnudas Sarda^2^, Jerel Calzo^3^, Kendrin Sonneville^4^, Mihail Samnaliev^5^, S. Bryn Austin^2^

##### ^1^La Trobe University, Australia; ^2^Boston Children’s Hospital, USA; ^3^San Diego State University, USA; ^4^University of Michigan School of Public Health, USA; ^5^Harvard Medical School, USA

###### **Correspondence:** Laura Hart (l.hart@latrobe.edu.au)

Purpose: To examine the relationship of disordered eating behaviours and eating disorder (ED) diagnosis in young adults with health-related quality of life (HRQL) and to assess the presence of effect modification by gender.

Methods: In 2013, 9440 participants (18–31 years) in the US Growing Up Today Study reported: use of disordered eating behaviours (dieting, diet pills, laxatives, or vomiting to control weight; binge eating with loss of control) over the past year, plus lifetime history of ED diagnosis. The relative risk (RR) of less-than-full health (EQ-5D-5 L health utility score < 1) and of any impairment (score > 1 on EQ5D-5 L dimensions) were compared across participants with and without disordered eating or ED diagnosis, using cross-sectional multivariate regression controlling for confounders. The association between HRQL and disordered eating or ED diagnosis was assessed using multivariable linear regression with the subsample reporting less-than-full health. The presence of effect modification by gender was also examined.

Results: Disordered eating behaviours and ED diagnosis were associated with significantly increased risk of less-than-full health. A significant gender interaction was found for ED diagnosis; men who reported ever having received a diagnosis experienced worse decrements in HRQL than did women. Inclusion of BMI in estimation models revealed small attenuations.

Conclusions: Disordered eating behaviours and ED diagnosis are associated with significant decrements in HRQL among young adult men and women.

### O51 Gay Bodies Worldwide: A 5-year prospective study of eating disorder and body image phenomena among sexual minority men

#### Scott Griffiths (scott.griffiths@unimelb.edu.au)

##### University of Melbourne, Australia

Gay Bodies Worldwide (*N* = 8200 [and growing]) is a 5-year prospective study of eating disorder and body image phenomena among sexual minority men. Starting in mid-February, a 45-day advertising campaign targeting millions of Grindr users was launched nationwide in Australia, Canada, the United Kingdom, and the United States. Design features of Gay Bodies Worldwide include 10 measurement occasions at 6-month intervals (ending in 2024), 2 modes of follow-up (SMS and email), and inclusion of items designed to augment missing data recovery. This talk will cover preliminary demographic data about the participants in Gay Bodies Worldwide, with emphasis given to demographic associations with eating disorder symptoms and diagnoses. As the largest dedicated longitudinal study of sexual minority men in the abovementioned countries (and potentially the world), Gay Bodies Worldwide has significant potential to uncover risk factors, novel mechanisms, and intersectionalities in the context of eating disorder and body image phenomena among sexual minority men.

### O52 Prevalence and features of eating disorders amongst first Australians

#### Phillipa Hay^1,2^, Adam Burt^1^

##### ^1^School of Medicine, Australia; ^2^Translational Health Research, Institute Western Sydney University, Australia

###### **Correspondence:** Phillipa Hay (p.hay@westernsydney.edu.au)

This study aimed to establish the prevalence of eating disorders amongst First Australians and to compare clinical features and health related quality of life (HRQoL) in First and other Australians with and without an eating disorder. Methods: Data were sourced from the general population 2015 and 2016 interview-based Health Omnibus surveys in South Australia. Eating disorder questions were based on the Eating Disorder Examination and Health Related Quality of Life (HRQoL) measured with the Short-Form 12 v1. Results: Twenty-five percent of the 93 First Australian survey respondents had an eating disorder which was significantly more than other Australians with an eating disorder (*p* = .04). Five percent had full threshold eating disorders (bulimia nervosa or binge eating disorder), and the remainder an Other (6%) or Unspecified (14%) Feeding or Eating Disorder (O/UFED- most with an eating disorder characterised by recurrent binge eating). First Australians with an eating disorder had higher levels of overvaluation than all other groups. They were also younger and had poorer Mental HRQoL than other Australians without an eating disorder. On binary regression, age and Mental HRQoL emerged as significant explanatory variables for the increased rate of eating disorders. Conclusions: Eating disorders are very common in First Australians and are associated with particularly high levels of overvaluation.

### O53 New population-based information on lesser studied eating and body image disorders in adolescence: Night eating syndrome and muscle Dysmorphia

#### Deborah Mitchison^1,3^, Jonathan Mond^2^, Kay Bussey^3^, Nora Trompeter^3^, Alexandra Lonergan^3^, Phillipa Hay^1^

##### ^1^School of Medicine, Translational Health Research Institute, Western Sydney University, Australia; ^2^Centre for Rural Health, University of Tasmania, Australia; ^3^Centre for Emotional Health, Department of Psychology, Macquarie University, Australia

###### **Correspondence:** Deborah Mitchison (deborah.mitchison@westernsydney.edu.au)

Background**:** There remains scarce population-based information on several eating disorders and body image disorders that may be more relevant to male adolescents, including night eating syndrome and muscle dysmorphia. The aim of this study was to assess the prevalence, demographic distribution and burden of these two disorders.

Method: Data comes from Wave 1 of the EveryBODY Study, a cross-sectional survey of body image and disordered eating in 5191 Australian adolescents aged 12–19 years. Participants completed questions on demographic characteristics, symptoms of night eating syndrome and muscle dysmorphia, quality of life, psychological distress, and weight and height.

Results: 177 (4.1%) participants met criteria for night eating syndrome, and prevalence did not differ by gender, weight status, migrant status, or socioeconomic status. However adolescents in grades 9–10 were twice as likely to meet criteria for night eating syndrome than participants in grades 7–8. Night eating syndrome was associated with quality of life impairment and elevated levels of psychological distress. Operationalising muscle dysmorphia was relatively more complicated and the prevalence (0.9–2.5%), distribution and burden of muscle dysmorphia varied considerably depending on the criteria applied.

Discussion: night eating syndrome and muscle dysmorphia are common disorders during adolescence that impact both boys and girls. Research criteria for muscle dysmorphia should consider where and how to draw the distinction with an eating disorder.

### O54 Carer Peer Mentor Pilot Program

#### Catherine Doyle^2^, Jacqueline Byrne^1^, Amy Hannigan^1^, Belinda Chelius^2^, Carmel Fleming^1^

##### ^1^Queensland Eating Disorder Service, Australia; ^2^Eating Disorders Queensland, Australia

###### **Correspondence:** Catherine Doyle (admin@edq.org.au)

Eating Disorders Queensland and theQueensland Eating Disorders Service work closely with carers on a daily basis. We recognise that eating disorders have significant effects on families as well as individuals (Zucker, Marcus & Balik, 2006). In 2019 the two organisations jointly rolled out a pilot Carer Peer Mentor Program in Queensland. The program connected and matched carers currently supporting loved ones with eating disorders to carers who had previously supported a loved one to recovery. Carers can be impacted by a range of personal, social, occupational and economic difficulties (Goodier et.al, 2013). They can experience a high burden of care (Whitney et al. 2005) and often feel isolated and excluded (Fox, Dean & Whittlesea 2017). Peer support recognises the value of lived experience in a meaningful and compassionate way. The aim of the pilot was to provide support for carers through a structured and supervised peer mentor program. Evaluation will include quantitative and qualitative analysis of implementation, feasibility, acceptability and impact of the program to assist in continuous improvement in the area of carer need as we grow and expand the program in Queensland.

### O55 Enlisting carers in adult eating disorders treatment

#### Belinda Caldwell^1^, Joshua Watson^2,3^

##### ^1^Eating Disorders Victoria, Australia; ^2^CEED, Australia; ^3^Eastern Health, Australia

###### **Correspondence:** Belinda Caldwell (belinda.caldwell@mh.org.au)

There is an emerging evidence base and policy drivers for working with carers in adult eating disorders treatment, and yet families/carers experience high diversity in the experience of inclusion with services and clinicians. Effectively harnessing families/carers around the person with the eating disorder can provide supportive recovery environments which can enhance transition to home planning, effective outpatient treatment and reduce hospital admissions. The Victorian Centre of Excellence in Eating Disorders undertook a state-wide project including a literature review, consulting with adult services and clinicians, and surveying families/carers, to inform the development of clear framework and specific guidelines for services and clinicians. This presentation will outline the outcomes of this project, the outcomes from a workshop undertaken with clinicians and services working in the adult treatment space and provide an overview of the guidelines. The guidelines cover the rationale for inclusion of families/carers, the challenges including confidentiality and interpersonal family interactions, and practical considerations in developing carer inclusive treatment plans.

### O56 The integration of digital technologies into the health care pathways for people with eating disorders

#### Helen Rydge^1^, Sarah Maguire^1,2^, Ellie Rogers^1^, Sean Rom^1^, Sarah Barakat^2^, Danielle Maloney^2^, Stephen Touyz^2^, Jane Miskovic-Whealtey^2^

##### ^1^InsideOut Institute, Australia; ^2^University of Sydney, Australia

###### **Correspondence:** Helen Rydge (helen@insideoutinstitute.org.au)

Clients with eating disorders often don’t receive treatment until years after the onset of illness, for a range of reasons including stigma, ambivalence, cost, and access. The eMental health space is growing exponentially and offers unique opportunities for reach, scale, automation, access and data capture. By developing eMental health technologies for eating disorders we can meet our clients where they are, offer tangible supports to a resource challenged health system, help overcome barriers to access and treatment and automate the reporting of health outcomes. The evidence base for the use of technical innovations in eating disorders will be overviewed and the InsideOut Institute’s eClinic presented. Data from the online screening and assessment tools, CBT eTherapy and eLearning hub for health professionals will be presented, which demonstrate significant impacts of digital technology both in treatment delivery and workforce training. Participants in our eTherapy pilot exhibited significant changes in core illness features pre to post intervention, and following elearning clinicians show significant improvements in all key learning concepts. The limitations of the eSpace in health care provision, the importance of integration of digital components with traditional care models and the impact of digital disruptors on the health care system will be discussed.

### O57 Evaluating treatment outcomes for eating disorders using a multidisciplinary, intensive, stepped-care approach

#### Kim Hurst^1^, Vinay Garbharran^2^, Melissa Marks^2^, Lauren Dasey^3^, Aimee Maxwell^2^

##### ^1^Eating Disorder Day Program, Australia; ^2^Robina Private Hospital, Australia; ^3^Mental Health, Gold Coast Hospital, Australia

###### **Correspondence:** Kim Hurst (kim.hurst@healthecare.com.au)

Day hospital treatment for eating disorders provides an alternative to an inpatient admission, it can be used as step-up when progress in outpatient therapy is insufficient or step-down following an inpatient admission. Improvements have been shown to result in reductions in remission rates, inpatient bed days and overall costs of treatment. The Robina Private Hospital Adolescent and Adult Eating Disorder Day Program has been operating since May 2017, consisting of a multidisciplinary team of psychologists, dietitian, psychiatrist and art therapist. The program provides intensive daily care (group and individual treatment) and allows patients to maintain some degree of educational, vocational and family life, while also giving an opportunity to practice coping skills resulting in reduced dependency, control issues, and the stigma associated with psychiatric hospitalisation. The program utilises a combination approach of cognitive behaviour therapy, interpersonal therapy, self-compassion therapy, dialectical behaviour therapy skills, art therapy and cognitive remediation. Measures collected included the Eating Disorder Examination Questionnaire, the Rosenberg Self-Esteem Scale, Difficulties in Emotional Regulation Scale, Multidimensional Perfectionism Scale, Experiences in Close Relationships, The Compulsive Exercise Test, and Stages of Change Questionnaire. Data was analysed using hierarchical linear modelling, analysis of variance, and one-way t-tests. Results indicated significant weight gain in underweight patients from pre-to-post, a significant reduction in global EDE-Q scores, and significant change in perfectionism scores.

### O58 Getting ready for a future that recognises competence in eating disorder treatment

#### Hilary Smith, Beth Shelton

##### National Eating Disorders Collaboration, Australia

###### **Correspondence:** Hilary Smith (hilary.smith@nedc.com.au)

This is a time of significant change in the treatment of eating disorders across Australia. On the eve of the introduction of new Medicare items to which Mental Health Professionals, Dietitians, Paediatricians and General Practitioners will need to be ready to respond, this session introduces the transdisciplinary competencies that are needed to effectively respond to eating disorders in community-based care settings. The National Eating Disorders Collaboration (NEDC) published the National Practice Standards for Eating Disorders in 2018. The standards include a competency framework for safe and effective identification of and response to eating disorders. This trans-disciplinary tool articulates the skills and knowledge requirements for practitioners at any point in the eating disorders continuum of care, from early identifier and initial responder through to shared care and treatment clinicians and recovery support. This presentation will outline the competency framework and the different roles that practitioners may be required to play within a multi-disciplinary team. The presentation will explore the relevance of the competence standards to the new Medicare items and how the NEDC tools may be used to support practice and professional development planning.

## Posters

### P1 Carers and clinicians: Partnering for quality professional development

#### Belinda Caldwell^1^, Gordon Brockway^2^

##### ^1^Eating Disorders Victoria; ^2^Eating Disorders Families Australia

###### **Correspondence:** Belinda Caldwell (belinda.caldwell@edfa.org.au)

In February and March 2019, the peak national body for families and carers, Eating Disorders Families Australia (EDFA), collaborated with the Victorian Centre of Excellence in Eating Disorders, Queensland Eating Disorders Service, InsideOut Institute, the South Australia Statewide Eating Disorders Service and Western Australian Eating Disorders Outreach and Consultations Service to run workshops and training across Australia on a new model of treatment, Temperament Based Therapy with Supports. We also worked collaboratively with ANZAED to promote the workshops, offer discounts for members, and with EDANZ, our equivalent organisation in New Zealand.

This initiative drew upon clinical and lived experience voices and resources to bring out the developers of the model from the USA, provide workshops for clinicians and carers and 4 day training for clinicians. The benefit for EDFA was that the model is evidence informed and practically engages carers/supports in the treatment model for adult eating disorders. The benefit for clinicians was access to excellent training at a reasonable cost, with some state partners also offering scholarships. Over 350 people attended all workshops/training, with 150 of those being clinicians.

The presentation will focus on the evaluations from the workshops, how clinicians and carers experienced learning together and whether a joint venture approach in professional development enhances the sector.

### P2 Understanding treatment access for eating disorders: Systems thinking as a complementary paradigm

#### Ben R Lane^1^, Lesley Cook^2^, Gemma J.M. Read^1^, Paul M. Simon^1^

##### ^1^University of the Sunshine Coast; ^2^Partners in Practice

###### **Correspondence:** Ben Lane (blane@usc.edu.au)

Patient and clinician decisions are shaped by the eating disorder treatment system within which they operate. For example, resource allocations and organisational rules constrain choices of how, when, and what to treat. Increased Medicare funding is approaching, but it remains unknown how this change will interact with other system components, such as care coordination and diagnostic pathways, to influence nationwide outcomes. This presentation will introduce systems thinking and argue that it can provide insight into challenges surrounding treatment access. Specifically, the AcciMap method was used to categorise factors that influence access across six hierarchical treatment-system levels, covering individuals and clinicians through to organisations and government. Barriers and facilitators were identified from the peer-reviewed literature and mapped onto the AcciMap. The analysis demonstrated that, although many barriers and facilitators have been identified, little is known about how these factors interact to influence access. The ways in which factors at higher levels of the system integrate with the day-to-day operations and activities of clinicians, families, and people with eating disorders also requires assessment. Systems-thinking methods, such as AcciMap, can support the identification of complex interactions, assisting the development of interventions to optimise system performance. The presentation will further discuss how the systems-thinking paradigm is being explored through the Sunshine Coast Eating Disorder Access Trial to analyse the downstream effects of system changes.

### P3 On the path to group-based services: Canberra hospital adult inpatient eating disorder service

#### Eleni Pavlidou^1^, Claire Speer^2^, Dr. Ashwin Swaminathan^3^

##### ^1^Acute Allied Health Services Psychology Department, Canberra Health Services; ^2^Acute Allied Health Services Nutrition Department, Canberra Health Services; ^3^Canberra Health Services

###### **Correspondence:** Claire Speer (claire.speer@act.gov.au)

Background and Aim: Following the implementation of a standardised-care pathway for our inpatients, our multidisciplinary team identified opportunities to further improve our patients’ health care experience. Working within the availabilities of our acute general medicine ward setting we targeted evidence-based approaches to actively engage our patients in their ongoing recovery.

Methods: To increase access to holistic supportive care and break the social isolation within an acute setting, we commenced group-based lunches addressing supported weight increase via nursing supervision. Further, we introduced group therapy sessions using cognitive remediation training to improve practicing distress tolerance skills with others. Sessions and services provided were evaluated by patients, based on the Maudsley Eating Disorder approach, and by nursing staff using a specifically designed questionnaire including a combination of likert scale and open-ended questions.

Results: Post session patient evaluations reported both group mealtimes and therapy sessions within an acute medical setting having a positive impact on their perceived support to manage social isolation. Further, staff reported improved confidence to support patients and skill development associated with providing new model of group-based supportive care.

Conclusion: Providing opportunities to accommodate active engagement in a controlled environment aided further participation towards recovery and promoted social re-integration. Improved patient care experience also positively impacted staff involvement and promoted insight into their own skill development which can promote improved health care services and patient outcomes.

### P4 Challenges and successes in implementing a multidisciplinary assessment clinic in Singapore- KK Hospital’s experience

#### Courtney Davis^1^, Kumudhini Rajasegaran^1^, Amerie Sin Mi Baeg^2^

##### ^1^KK Hospital for Women and Children, Singapore; ^2^KK Women and Children’s Hospital Psychology Service, Singapore

###### **Correspondence:** Courtney Davis (courtney.davis@kkh.com.sg)

KK Hospital in Singapore has had an eating disorder treatment program since 2010 as part of the Adolescent Medicine Service. The service consists of a multidisciplinary care team of adolescent medicine physicians, psychologists, nutritionists, nurses, psychiatrists and social workers. The program started offering family based therapy (FBT) to all our patients in 2013. In review of our quality of eating disorder care, we identified some difficulties in our outpatient initial assessment phase including the need for multiple separate visits, families needing to review the history of presentation multiple times, and a time lapse between initial medical assessment and psychology assessment. To improve the quality of care, we have embarked on instituting a multidisciplinary assessment clinic, integrating physician, psychologist, and nutritionists assessments in one visit. While multidisciplinary assessment clinics have been widely used in other healthcare systems, implementing an eating disorder assessment clinic in the Singapore health care context has posed some unique challenges including appropriate identification and triaging of cases, costing, and communicating the nature of the clinic and cost to families. This poster summarizes the process evaluation of our multidisciplinary assessment clinic implementation.

### P5 Achieving early identification and intervention in the performing arts

#### Jessica Ryan^1,2^, Alexandra Hillman^1,3^, Beth Shelton^4^, Emma Spiel^1^

##### ^1^CEED; ^2^Melbourne Health; ^3^NorthWestern Mental Health; ^4^NEDC

###### **Correspondence:** Beth Shelton (beth.shelton@icloud.com)

Elite vocational performance training is recognized as an increased risk for eating disorders (ED). The University of Melbourne (UoM) Victorian College of the Arts (VCA) is Victoria’s leading provider of tertiary training for professional artists. The VCA Dance and Music Theatre Faculties and the Victorian Centre of Excellence in Eating Disorders (CEED) collaborated to clarify and develop a co-responsibility model to assist in the identification of EDs in adult students. The project aimed to maximize the potential for early identification and early, evidence-based treatment. This paper will outline an early intervention process of change within this high risk population. It will draw upon qualitative data obtained from students and staff, and explore the implications of these in the development of the current project and future early identification projects. The paper will describe the strategic interventions which were developed and implemented as part of the project. It provides insights into the culture and barriers for seeking support for EDs within the arts, and explores the process of change through collaboration and co-responsibility of students, staff and the broader university.

### P6 Neural response to low energy food images in anorexia nervosa

#### Nasim Foroughi^1^, Brooke Donnelly^2,3^, Mark Williams^4^, Michael Kohn^5^, Simon Clarke^5^, Perminder Sachdev^6^, Stephen Touyz^3^, Sloane Madden^3,7,8^, Phillipa Hay^9^

##### ^1^Western Sydney University; ^2^RPA Hospital; ^3^University of Sydney; ^4^Macquarie University; ^5^Westmead Hospital; ^6^Centre for Healthy Brain Ageing (CHeBA), University of New South Wales; ^7^The Sydney Children’s Hospital Network; ^8^The Redleaf Practice; ^9^School of Medicine and Centre for Health Research Western Sydney University

###### **Correspondence:** Nasim Foroughi (n.foroughi@westernsydney.edu.au)

This study compared neural responses to images of low-energy foods in patients with Anorexia Nervosa (AN) and an age-matched Healthy Control (HC) group. Women (age ≥ 14 yrs) with current AN (*n* = 25) and HCs without an eating disorder (*n* = 21) were recruited and underwent Functional Magnetic Resonance Imaging (fMRI) brain imaging, during which they viewed food and non-food (neutral) images. Images were chosen to evoke responses of disgust, happiness, or fear. All participants completed self-report questionnaires including eating disorder symptoms (the Eating Disorder Examination Questionnaire) anxiety and / or mood symptoms (The Hospital Anxiety and Depression Rating Scale). There were significant differences between the groups in the following areas: (1) people in the AN group showed more responsivity to low-energy food images in the frontal lobe (sub-gyral, superior, and middle frontal gyral), parietal lobe (supra-marginal gyrus), and one temporal area (sub-gyral) compared to the HC group; (2) greater activity was observed in the occipital lobe (visual processing), cerebellum, and temporal lobes in the HC vs. AN group. People without eating disorders may have a better capacity to filter salient from non-salient information relating to the current task. In summary, for those with AN, it would seem their ability to efficiently ‘sort-out’ information (especially information pertaining to disorder-relevant stimuli such as food images) in order to complete the task at hand, is diminished.

### P7 Withdrawn

### P8 Highlights and challenges of a dietitian working in an eating disorder outpatient day program

#### Lauren Dasey (lauren.dasey@healthecare.com.au)

##### Mental Health, Gold Coast Hospital

Although working as a dietitian in a hospital inpatient setting has its’ challenges, it can start to feel familiar and safe. Members of the multi-disciplinary team have a clearly defined role and work collaboratively with the patient, to maintain safety while working towards treatment goals. I was particularly familiar with a secure ward environment, where patients were often provided with 24 h individual nursing support.

When I commenced a new job in an eating disorder outpatient day program, I was excited by the opportunity to have more time with clients, to provide nutrition education in a group setting. The new role and environment provided highlights, however, it also presented some challenges that required constant self-reflection, clarification of roles and responsibilities, as well as ongoing development of skills and confidence.

Highlights included:

- co-facilitation with disciplines such as clinical nurse, psychologist and art therapist;
- the opportunity to complete weekly behavioural experiments at local cafes;
- longitudinal observation of behaviour change, impacting on quality of life

Challenges included:

- medical and psychological risk within group and during social eating outings
- interfering behaviours
- providing supportive meal therapy

This presentation will discuss these in more detail, including strategies used to address the challenges.

### P9 The effectiveness of expressive art therapy in an eating disorder day program setting

#### Melissa Marks^1^, Kim Hurst^2^, Lauren Dasey^3^, Vinay Garbharran^1^, Aimee Maxwell^1^

##### ^1^Robina Private Hospital; ^2^Eating Disorder Day Program; ^3^Mental Health, Gold Coast Hospital

###### **Correspondence:** Melissa Marks (melissa.marks@healthecare.com.au)

Expressive art-therapy combing psychology and creative processes is increasingly being added as an adjunct to traditional eating disorder therapies. The patient and the art-therapist work together to understand and derive meaning from symbols and images created, to promote emotional growth and healing by tapping into both the conscious and sub-conscious mind. Eating disorder behaviours are often used to reduce negative affect, escape painful feelings by numbing, and suppressing difficult or traumatic experiences. When the brain is starved, cognitive therapies may be less effective and the process of art-making can become a means to communicate, describe or depict thoughts, feelings and emotions without the use of words. It allows patients to safely explore themes that may be maintaining eating disorder behaviours by providing another medium to express themselves other than through a maladaptive relationship with food and their bodies. Using metaphoric language also assists clinicians to use the language of the patient rather than their own. Expressive art-therapy is an important component of treatment offered within the Robina Private Hospital’s eating disorder day program, it has assisted clinicians within the team to garner additional information or insight into the patient experience, and for patients, it complements therapeutic content, thus enhancing patient outcomes. This presentation will describe qualitative outcomes of how art-therapy was considered effective in an eating disorder day program setting.

### P10 Comparing DBT and RO-DBT as treatments for eating disorders

#### Tanya Gilmartin (tanyalgilmartin@gmail.com)

##### RO-DBT Australia

Dialectical Behaviour Therapy (DBT) was developed to treat disorders characterised by Emotion Dysregulation. The original program has been adapted to treat disordered eating patterns, based on an emotional dysregulation model of problem eating. DBT for Binge eating and bulimia has been found to effectively reduce problematic eating behaviour, and has been found to be particularly helpful for clients presenting with complex and co-morbid presentations. In contrast, Radically Open DBT (RO-DBT) was developed to treat disorders on emotional over-control, and and focuses on targeting temperamental biases and social signalling. Research has indicated that RO-DBT is an effective treatment for anorexia nervosa in both inpatient and outpatient settings. This presentation will compare and contrast the two treatments, and review the existing research literature. In particular, we aim to highlight the different theoretical orientations of the two treatments, and how these relate to treatment targets, structure and outcomes.

### P11 Introducing RO-DBT as a treatment for anorexia nervosa

#### Tanya Gilmartin (tanyalgilmartin@gmail.com)

##### RO-DBT Australia

Radically Open Dialectical Behaviour Therapy (RO-DBT) has been developed over 20 years based on clinical research designed to treat disorders characterised by emotional Over-control. It is theorised that individuals who constrict their emotional experience become isolated from their peers and that this, in turn, contributes to the Psychiatric difficulties that lead to them presenting to treatment. As a result, RO-DBT targets bio-temperamental and social signalling biases in a combination of group skills classes and individual therapy to assist clients in improving their social connectedness and therefore reducing their Psychiatric symptoms. This approach has been found to be an effective treatment for anorexia nervosa in both inpatient and outpatient settings, as it targets personality factors that were likely to be present prior to the development of restrictive eating, rather than the eating behaviour itself. This presentation aims to introduce RO-DBT as a treatment for anorexia nervosa by discussing the relevant underlying theory and treatment interventions, as well as reviewing the relevant research literature.

### P12 Temperament in treatment: Exploring a shift in treatment interventions for anorexia nervosa

#### Tanya Gilmartin (tanyalgilmartin@gmail.com)

##### RO-DBT Australia

Anorexia nervosa is considered a serious Psychological disorder which can have a significant negative impact on the health of the client. However, research into existing treatment approaches for adults suffering from the disorder have yielded mixed results. Past research into factors that may contribute to a cause of anorexia has indicated that the temperament of an individual with anorexia can be characterised by high state anxiety, high harm avoidance, low reward sensitivity, high self-control and a tendency toward detailed-focused processing. Additional neurological research has identified that these temperamental traits have been linked to increased brain activity in some areas, further suggesting that these traits are relatively stable throughout an individual’s lifespan. This recent neurological research has provided the framework for the development of new treatments that focus on managing underlying factors that have been found to precede the development of the disorder and impeded treatment prognosis. The current paper provides an overview of two such treatments, Temperament Based Therapy with Supports (TBT-S) and Radically Open Dialectical Behaviour Therapy (RO-DBT) which both take different approaches to the management of temperament in regard to anorexia nervosa.

### P13 Cardiometabolic profile of anorexia nervosa

#### Zoe Jenkins^1^, Andrea Phillipou^1^, David Castle^2,3^, Elisabeth Lambert^1^, Nina Eikelis^1^

##### ^1^Swinburne University of Technology; ^2^University of Melbourne; ^3^St Vincent’s Hospital

###### **Correspondence:** Zoe Jenkins (zoe.jenkins@svha.org.au)

The energy deprivation and malnutrition associated with Anorexia Nervosa (AN) places immense pressure on the cardiovascular system, with up to 80% of patients suffering from cardiovascular or metabolic complications, of which the aetiology remains poorly understood. Research suggests that cardiovascular and metabolic abnormalities are linked to disturbances in nerve activity called autonomic dysfunction. This research is specifically investigating sympathetic nervous system aberration in AN and its relationship to organ damage and metabolic abnormalities by measuring autonomic function in individuals with AN. It is hypothesised that the AN group will demonstrate atypical sympathetic nervous system compared to the control group, and this will be related to increased cardio-metabolic disturbances. Participants will include 30 individuals with AN, 30 recovered from AN and 30 matched controls who are assessed on measures of autonomic nervous system function including; nerve activity, endothelial function, arterial stiffness and sudomotor function. Participants provide blood and urine samples which undergo lipidomic and metabolomic analysis. The findings of this research has important implications for our understanding of the underlying causes of cardiovascular and metabolic abnormalities present in AN, and will ultimately have the potential to identify and prevent cardiovascular and metabolic damage in these individuals.

